# Longitudinal sequencing of cardiometabolic multimorbidity among older adults and association with subsequent dementia onset

**DOI:** 10.1371/journal.pone.0326309

**Published:** 2025-07-10

**Authors:** Corey L. Nagel, Siting Chen, Heather G. Allore, Anda Botoseneanu, Jason T. Newsom, Nick Bishop, David A. Dorr, Jeffrey Kaye, Ana R. Quiñones

**Affiliations:** 1 College of Nursing, University of Arkansas for Medical Sciences, Little Rock, Arkansas, United States of America; 2 Department of Biostatistics, College of Public Health, University of Arkansas for Medical Sciences, Little Rock, Arkansas, United States of America; 3 OHSU-PSU School of Public Health, Oregon Health & Science University, Portland, Oregon, United States of America; 4 Department of Internal Medicine, School of Medicine, Yale University, New Haven, Connecticut, United States of America; 5 Department of Biostatistics, School of Public Health, Yale University, New Haven, Connecticut, United States of America; 6 Department of Health & Human Services, University of Michigan, Dearborn, Michigan, United States of America; 7 Institute of Gerontology, University of Michigan, Ann Arbor, Michigan, United States of America; 8 Department of Psychology, Portland State University, Portland, Oregon, United States of America; 9 Norton School of Human Ecology, University of Arizona, Tucson, Arizona, United States of America; 10 Department of Medical Informatics and Clinical Epidemiology, Oregon Health & Science University, Portland, Oregon, United States of America; 11 Department of Neurology, Oregon Health & Science University, Portland, Oregon, United States of America; 12 Department of Family Medicine, Oregon Health & Science University, Portland, Oregon, United States of America; Albert Einstein College of Medicine, UNITED STATES OF AMERICA

## Abstract

**Background:**

Patterns of development of cardiometabolic multimorbidity (CMM) and the impact of specific cardiometabolic disease combinations on cognitive function are not well understood. This study utilizes sequence analysis to describe the ordering and timing of cardiometabolic disease accumulation over a five-year period and to assess both sociodemographic predictors and cognitive outcomes of typical cardiometabolic disease sequences.

**Methods:**

We analyzed data from the National Health and Aging Trends Study (2011–2022), including respondents aged ≥65 years without CMM or cognitive impairment at baseline (N = 4956). We used sequence analysis with optimal matching and hierarchical cluster analysis to describe temporal patterns of cardiometabolic disease accumulation and to construct a typology by clustering similar sequences. Sociodemographic predictors of CMM cluster membership were assessed using multinomial logistic regression and discrete time survival analysis was used to examine the association of CMM clusters with subsequent dementia development.

**Results:**

11.8% of respondents developed CMM within 5-years. From a total of 366 distinct cardiometabolic disease sequences, we identified eight cardiometabolic sequence clusters. The first five clusters, “*No Cardiometabolic Disease*” (N = 2283, 46.1%); “*Diabetes Only*” (N=642, 13.0%); *Heart Disease Only*” (N = 297, 6.0%); “*MI Only*” (N = 145, 2.9%); “*Stroke Only*” (N = 132, 2.7%), were composed of persons who did not develop CMM over the observation period. The sixth cluster, “*Incident CVD with Multimorbidity*” (N = 656, 13.2%), was largely composed of persons with no conditions at baseline who developed incident cardiometabolic disease and/or CMM during the observation period (N = 477, 72.7%) and the seventh cluster, “*Diabetes Multimorbidity*” (N = 333, 6.7%), primarily consisted of persons with diabetes who developed incident CMM. Finally, the eight cluster (N = 468, 9.4%) was characterized by mortality early in the observation period with minimal CMM development during the observation period. Black and Hispanic race/ethnicity, lower wealth, and obesity were associated with increased likelihood of membership in one or both of the clusters characterized by CMM development. We observed increased dementia risk among persons in the *Incident CVD with Multimorbidity* cluster (HR = 1.32, 95% CI = 1.04–1.67) and the *Diabetes MM* cluster (HR = 1.88, 95% CI = 1.44,2.44).

**Conclusions:**

Development of cardiometabolic multimorbidity is more likely among minoritized and/or low-income older adults and is associated with increased risk of subsequent dementia. Targeted approaches to cardiometabolic disease prevention and risk reduction may be an effective means of slowing or preventing the onset of cognitive decline among these groups.

## Introduction

The prevalence of multimorbidity, defined as the co-occurrence of two or more chronic conditions, has steadily increased both in the US and globally over the last two decades [1–3]. Associated with declines in physical and cognitive function [4–6], increased health care utilization [7,8], decreased quality of life [9], and increased mortality [10], multimorbidity poses a substantial and growing challenge to individuals and their families. However, despite an increasing body of research into multimorbidity, the majority of studies continue to examine multimorbidity in terms of the number of conditions present [11], most commonly operationalized as an unweighted or weighted count (such as the Charlson comorbidity index [12]). Fewer studies assess the risks to function, maintenance of independence, and health-related quality of life associated with specific multimorbidity combinations. Further, to date, there have been few studies that explicitly focus on the temporal sequencing and patterning of disease occurrence in multimorbidity development, despite a general understanding that differences in the timing, ordering, and presence of specific disease combinations result in substantial variation in multimorbidity-associated outcomes [13].

The paucity of studies that examine multimorbidity in the context of both specific disease combinations, what Prados-Torres et al. refer to as “associative multimorbidity,” and the longitudinal characteristics of timing and ordering of disease development, can be partly attributed to the methodological complexity involved [14]. There are few approaches commonly used in the health sciences suited to examining longitudinal patterns of discrete events occurring over time and their combinations. A promising, though underutilized, approach to longitudinal multimorbidity research is the method known as sequence analysis. First developed for genetic research, it has been widely adopted in the social sciences to describe and analyze series of events occurring over time [15]. However, it is uncommon in medical or public health research, with only one recent study using sequence analysis to examine multimorbidity in Scotland over 20-years [16] and no studies to date have employed this approach to examine multimorbidity among older adults in the US.

Here we demonstrate the use of sequence analysis to examine the development and cognitive sequelae of cardiometabolic multimorbidity (CMM), defined as the co-occurrence of two or more cardiometabolic diseases (diabetes, heart disease, stroke, and myocardial infarction), among a nationally-representative sample of US older adults. The relationship between cardiovascular disease and dementia risk has long been recognized [17], and there has been increasing focus on the association of diabetes with dementia [18]. We focus on CMM because there is limited though increasing evidence that the risk of cognitive decline and dementia are greater in the context of multiple cardiometabolic conditions compared to single conditions [19–22]. For example, Jin et al. [19] reported a significant and dose-dependent association between increased number of cardiometabolic conditions and declining cognitive function among a pooled cohort drawn from the Health and Retirement Study and three harmonized international cohorts. Similarly, Khondoker et al. [20] used latent class analysis to identify multimorbidity clusters among a large cohort of older adults in the UK Biobank and reported that multimorbidity patterns dominated by cardiometabolic conditions were associated with a doubling of the risk of dementia. We advance the previous work in this area by using a novel approach to examine distinct longitudinal patterns of CMM development and their differential relationship to dementia risk. Further, as there is strong and consistent evidence among US older adults that both multimorbidity and dementia risk are socially patterned and are disproportionately experienced by low-income and racial/ethnic minoritized persons [23–26], we explicitly focus on racial/ethnic differences in CMM as a potential factor contributing to observed disparities in cognitive decline and dementia risk. Findings from this study may support potential targets for clinical preventive and therapeutic interventions aimed at reducing the cognitive burden of cardiometabolic conditions and reducing social disparities in cognitive outcomes in later life

## Methods

### Data source

We analyzed data from participants in the National Health and Aging Trends Study (NHATS) during the years 2011–2021. The NHATS is an ongoing, nationally-representative cohort study of Medicare beneficiaries 65 years or older. The initial sample was enrolled for interviews in 2011, with a replenishment of the sample enrolled in 2015. Interviews are conducted annually and full details of NHATS have been published [27]. The NHATS protocol was approved by the Johns Hopkins University Institutional Review Board (IRB) and the study protocol was approved by our institutional IRB (STUDY00019414).

### Study sample

All community-dwelling respondents enrolled in either the 2011 or 2015 NHATS cohorts were screened for inclusion in the analytic sample (N = 11000). We first excluded respondents with a baseline cognitive score indicative of dementia (N = 1272). We then excluded persons with missing data on self-reported cardiometabolic conditions and/or cognitive status at all survey waves (N = 125). In order to calculate longitudinal sequences of CMM, we excluded respondents lost to follow-up due to non-mortality attrition within 5-years of their baseline NHATS wave (N = 3495). We did not exclude respondents who were lost to follow-up due to mortality, as death was included as an absorbing state in sequence analysis. Because the focus of analyses is on the development and progression of CMM, we excluded respondents with cardiometabolic conditions at baseline (N = 980). Finally, we excluded 172 respondents who had missing race/ethnicity data or reported “other” as their racial/ethnic category because of heterogeneity within this racial/ethnic identity group. This resulted in the ascertainment of five-year cardiometabolic disease sequences on 4956 respondents. Of those, 3652 remained in the NHATS cohort and were dementia free at the end of the five-year sequence ascertainment period and were included in the discrete time survival analysis of the association between cardiometabolic sequence group and dementia onset. A graphical depiction of the sample flow is provided as S5 Fig.

### Measures

The presence of cardiometabolic conditions was ascertained using four self- and proxy-reported, physician-diagnosed conditions (diabetes, heart disease, stroke, myocardial infarction (MI)) assessed at each annual NHATS interview. In the NHATS interview, heart disease is queried as “any heart disease including angina or congestive heart failure” and myocardial infarction is queried as “a heart attack or myocardial infarction” [28]. To resolve potential longitudinal inconsistency in the reporting of cardiometabolic disease status [29] and to remain consistent with the item wording-which asks whether a physician ever told the respondent that they have a given condition-we carried forward positive reports of index conditions over the duration of the study period.

Cognitive testing is performed annually in the NHATS cohort and is designed to evaluate several aspects of cognitive function [30,31]: 1) memory with an immediate and delayed 10-word recall test (range: 0–20); 2) orientation by querying the date, month, year, and day of the week and naming the President and Vice President (range: 0–8); and 3) executive function with a clock drawing test (range: 0–5). The total cognitive function score (range: 0–33) is derived by summing the scores from the composite subscales, with higher scores indicating better cognitive function. Based on the resulting score, individuals are categorized as “no dementia”, “possible dementia” or “probable dementia” using an established algorithm [32]. In the current analysis, we applied a conservative approach and defined dementia as a score which met or exceeded the score threshold for probable dementia.

We utilized sociodemographic information from respondents’ baseline interview, including age (in years), sex, race/ethnicity (mutually exclusive categories for non-Hispanic White, non-Hispanic Black, and Hispanic), educational attainment (less than high school education, high school graduate, some college, college graduate or greater), marital status (married/living with a partner) and reported household income. BMI was calculated at baseline according to the established formula (BMI = weight [pounds] x 703/ height^2 [inches]). Smoking status was dichotomized into any history of tobacco smoking or not. Baseline self-reported health was reported using a five-item Likert-type scale ranging from 1 (poor) to 5 (excellent). Individual items assessing reported difficulty in activities of daily living (ADL) and instrumental activities of daily living (IADL) were summed to yield a baseline count of ADL/IADL limitations at respondents’ baseline wave. The remainder of the self-reported conditions assessed by the NHATS (arthritis, lung disease, osteoporosis, hypertension, and cancer) were summed at each wave to yield a count of additional conditions at each time point. Finally, death information was gathered from informants during attempts to contact respondents for their annual NHATS interview.

### Statistical analysis

We examined five-year patterns of CMM progression using single-channel sequence analysis. Briefly, sequence analysis is a non-parametric approach used to evaluate the observed sequences of individuals’ progression through a series of defined states over time. These sequences can be characterized using descriptive statistics and data visualization techniques, as well as agglomerated into distinct groups sharing similar trajectories using cluster analysis or other dimension reduction techniques [33].

In sequence analysis, the sequence of states over time can be described in terms of 1) the occurrence of discrete states, 2) the ordering of those states, and 3) the duration spent in each state. Raw sequence data, where each state is represented visually or symbolically at each observed time point, are referred to as being in state sequence (STS) format. By aggregating adjacent and identical states over time, we can simplify the representation of sequences by removing the element of duration spent in each state to focus solely on the sequencing and order of states. This reduced format, known as distinctive successive states (DSS) format, places emphasis on the representation of state transitions (e.g., transitioning from diabetes to diabetes with heart disease). In describing the observed cardiometabolic sequences among the sample, we utilize both STS and DSS format as appropriate.

In this study, the four assessed cardiometabolic conditions and their potential combinations yielded a total of sixteen distinct states: (1) “none”, (2) “diabetes”, (3) “heart disease”, (4) “stroke”, (5) “myocardial infarction”, (6) “diabetes + heart disease”, (7) “diabetes + myocardial infarction”, (8) “diabetes + stroke”, (9) “heart disease + myocardial infarction” (10) “heart disease + stroke”, (11) “myocardial infarction + stroke”, (12) “diabetes + heart disease + myocardial infarction”, (13) “diabetes + heart disease + stroke”, (14) “diabetes + myocardial infarction + stroke” (15) “heart disease + myocardial infarction + stroke”, (16) “diabetes + heart disease + myocardial infarction + stroke”. Including an additional absorbing state of death yielded a total of 17 non-overlapping states that described respondents cardiometabolic disease status at each wave. The cardiometabolic disease history of each individual in the sample is thus represented by a sequence of six states, representing their reported disease status at baseline interview and annually thereafter for five years (2011–2016 for the 2011 cohort, 2015–2021 for the 2015 cohort).

Following the calculation of descriptive statistics and construction of plots to characterize the resulting sequences, we used optimal matching (OM) to cluster sequences into distinct groups based on pairwise comparison of constituent states, state sequencing, timing, and duration [34]. This approach calculates a value termed “distance” between each pair of observed sequences. Calculation of the distance is based on genetic concepts of the cost to transform one sequence to the other using a series of substitutions, insertions, or deletions [35]. Specifically, we used a constant substitution cost of 1 (essentially weighting state transitions equally), and an and an insertion/deletion value termed “indel” of 1. We selected these values in order to reflect our uncertainty in the relative importance of specific diseases (e.g. substitution cost of 1 results in weighting substitutions equally) and a neutral stance in regard to transition timing (indel = 1). Next, we applied hierarchical cluster analysis with Ward’s algorithm to the resulting matrix of calculated distances to generate a range of potential cluster solutions (range 2–12) and used goodness-of-fit indictors and visual comparison to identify the optimal cluster solution.

The results of the cluster analysis were used to categorize respondents into distinct cardiometabolic sequence groups based on similarity in disease patterns and timing. We calculated descriptive statistics to summarize the characteristics of the members of each sequence group and used multinomial logistic regression to identify sociodemographic factors associated with respondents’ membership in each sequence group. For those participants in the sample who were still living and had not developed dementia at the end of the five-year sequence ascertainment period, we examined the relationship of sequence group membership to the hazard of dementia onset during the subsequent NHATS waves using discrete-time survival analysis to account for both interval and right censoring. We used a binomial model with a complementary log-log link and incorporated a gamma-distributed frailty to account for unobserved individual heterogeneity. Modeling was conducted in two steps: first, an age-adjusted model estimated the association of cluster membership with subsequent dementia hazard, then a fully adjusted model included age, sex, race/ethnicity, education, baseline income, year of cohort entry (2011 vs 2015), and a time-varying count of non-cardiometabolic conditions. As the complementary log-log model is a proportional hazards survival model, we assessed the proportional hazards assumption by testing the significance of a time*multimorbidity cluster interaction. Sequence analysis was conducted in R using the TrajMinR [36], TraMineRextras [37], and ggseqplot [38] packages. Discrete time survival analysis was conducted in Stata 17 using the pgmhaz2 [39] package. The R code used to perform this analysis is provided in supporting information.

## Results

### Sample characteristics

Selected baseline sociodemographic and health characteristics of the analytic sample are provided in Table 1. The mean age of the sample at baseline was 76.2 years (SD = 7.5) and a majority were female (58.6%). Non-Hispanic White respondents were the largest (75.0%) racial/ethnic group, followed by non-Hispanic Black (19.9%) and Hispanic (5.1%) older adults. A slight majority (53.4%) of respondents reported some college or higher education and 19.8% reported less than high school education.

**Table 1:** General characteristics of analytic sample at baseline interview.

Characteristic	Mean (SD) or N (%)
**Age**	76.20 (7.50)
**Sex**	
* Male*	2,050 (41.4%)
* Female*	2,906 (58.6%)
**Race/Ethnicity**	
* Non-Hispanic White*	3,718 (75.0%)
* Non-Hispanic Black*	988 (19.9%)
* Hispanic*	250 (5.1%)
**Born in US**	4,571 (92.2%)
**Educational Attainment**	
* Less than high school*	982 (19.8%)
* High school graduate*	1,325 (26.7%)
* Some college*	1,061 (21.4%)
* College graduate*	1,588 (32.0%)
**Married/Partnered**	2,616 (52.8%)
**Household Income** (thousands)	56.19 (148.9)
**Diabetes**	903 (18.2%)
**Heart Disease**	390 (7.9%)
**Stroke**	205 (4.1%)
**Myocardial Infarction**	254 (5.1%)
**Number of Chronic Conditions**	2.19 (1.30)
**Number of Non-Cardiometabolic Conditions**	1.80 (1.10)
**Body Mass Index**	27.9 (5.7)
**Self-Rated Health**	
* Excellent*	801 (16.2%)
* Very good*	1,617 (32.6%)
* Good*	1,584 (32.0%)
* Fair*	757 (15.3%)
* Poor*	195 (3.9%)
**Number of ADL/IADL Limitations**	1.28 (2.24)
**Cognitive Score**	18.70 (4.50)

1Mean (SD); n (%); ADL = Activities of Daily Living; IADL = Instrumental Activities of Daily Living.

Overall, sample members reported a mean of 2.1 (SD = 1.3) chronic conditions, including both cardiometabolic and non-cardiometabolic disease, at study baseline. More than half (N = 3204, 64.7%) did not report any history of cardiometabolic disease at their baseline interview. Among the remaining 1752 (35.4%) respondents with a cardiometabolic condition at baseline, the most common reported condition was diabetes (N = 903, 18.2%), followed by heart disease (N = 390, 7.9%), myocardial infarction (N = 254, 5.1%), and stroke (N = 205, 4.1%). The average baseline cognitive score was 18.7 (SD = 4.5) and respondents reported an average mean of 1.2 (SD = 2.2) ADL/IADL limitations. Most of the sample members (80.8%) reported themselves to be in good to excellent health at baseline.

### Description of cardiometabolic disease sequences

Over the five-years sequencing assessment, 50.1% (N = 2484) of participants did not develop any cardiometabolic diseases, 38.1% (N = 1889) ended the study period with a single cardiometabolic disease, and 11.8% (N = 583) developed CMM. As expected, the development of CMM was more likely among those with an existing cardiometabolic disease at baseline, with 23.2% (N = 406) of respondents with an existing cardiometabolic disease at baseline developing one or more additional conditions (i.e., CMM) during the observation period. In contrast, among the 3204 respondents without a baseline cardiometabolic condition, 19.4% (N = 623) developed a single cardiometabolic disease and 5.5% (N = 177) developed CMM.

Among the sample, we observed a total of 366 unique STS sequences (in which the states occurring at each time point are represented). Because the visualization of many distinct sequences is challenging to interpret, we converted these to DSS format (in which only transitions to new states are represented) for visualization purposes. This reduces the number of distinct sequences from 366 to 126. (We provide a visual representation of the observed STS-format sequences for the full sample in S1 Fig).

**Fig 1:**
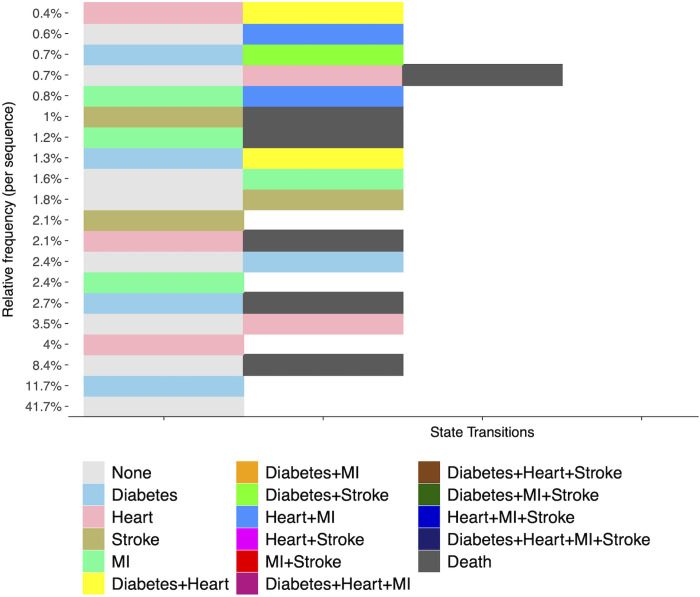
Relative frequency of 20 most common cardiometabolic disease sequences in reduced format. This sequence frequency plot displays the twenty most commonly occurring cardiometabolic disease sequences in distinctive successive states (DSS) format. DSS sequences display the temporal ordering of disease states and transitions without regard to duration. The proportion of respondents with each sequence (relative to the total sample) is displayed on the y-axis.

The twenty most common sequences in DSS format are visualized in Fig 1 and presented in tabular form in Supplemental Table 1. The most common sequence was characterized by no cardiometabolic disease across the observation period (41.8%), followed by diabetes present at baseline without development of cardiovascular disease (11.7%). The most common CMM sequence among the total sample was transitioning from diabetes at baseline to diabetes and heart disease (1.3% of total sample, 7.1% of those with diabetes at baseline).

Among persons with diabetes at baseline, 21.3% developed some combination of CMM during the observation period, with the most common multimorbidity sequence being baseline diabetes to diabetes and heart disease, as described above. Among respondents with heart disease at baseline, 22.8% transitioned to CMM, with the most common sequence being baseline heart disease to heart disease and diabetes (5.6%). Progression to CMM occurred among 25.4% of respondents with a history of stroke at baseline, with the most common sequence being stroke followed by stroke and heart disease (7.8%). Among respondents with MI at baseline, 28.7% progressed to CMM, with the most common sequence being MI to MI and heart disease (15.0%).

**Fig 2:**
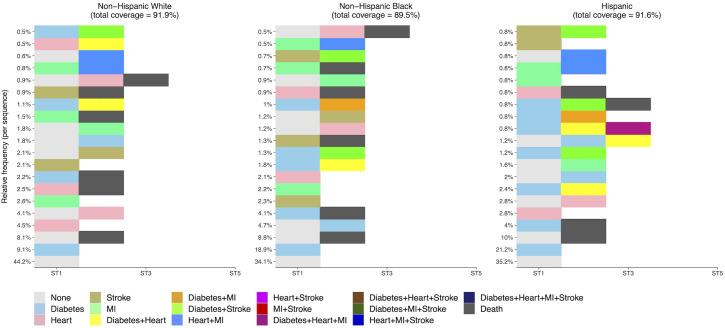
Relative frequency of most common cardiometabolic disease sequences stratified by racial/ethnic group. This plot displays the relative frequency of the twenty most commonly occurring cardiometabolic disease sequences in reduced (DSS) format within each racial/ethnic group. DSS sequences display the temporal ordering of disease states and transitions without regard to duration. The proportion of respondents with each sequence (relative to the total sample) is displayed on the y-axis.

The distribution of DSS sequences stratified by race/ethnicity is graphically depicted in Fig 2. The proportion of both non-Hispanic Black (34.1%) and Hispanic (35.2%) respondents who reported no cardiometabolic conditions during the study period was lower than that among Non-Hispanic White respondents (44.2%). Conversely, mortality occurring among individuals with no cardiometabolic conditions was slightly lower among non-Hispanic White respondents than among non-Hispanic Black or Hispanic respondents (8.1% vs 8.8% and 10%, respectively). Overall, CMM was more common among non-Hispanic Black and Hispanic respondents than among non-Hispanic White respondents (15.0% and 16.4% vs 10.6%). As the most prevalent cardiometabolic condition in the sample at baseline, diabetes was a substantial driver of observed racial/ethnic differences in CMM development. Compared with non-Hispanic White respondents, both Non-Hispanic Black and Hispanic respondents had higher proportions of diabetes alone (13.3% vs 28.1% and 27.2%, respectively) and diabetes with CMM (5.1% vs 10.6% and 12.0%). In contrast, heart disease in isolation was less common among both NH Black and Hispanic respondents than NH Whites, although heart disease in combination with other cardiometabolic conditions was slightly more common among non-Hispanic Black and Hispanic respondents than Non-Hispanic White respondents (9.9% and 10.8% vs 8.4%).

### Identification of cardiometabolic disease sequence clusters

**Fig 3:**
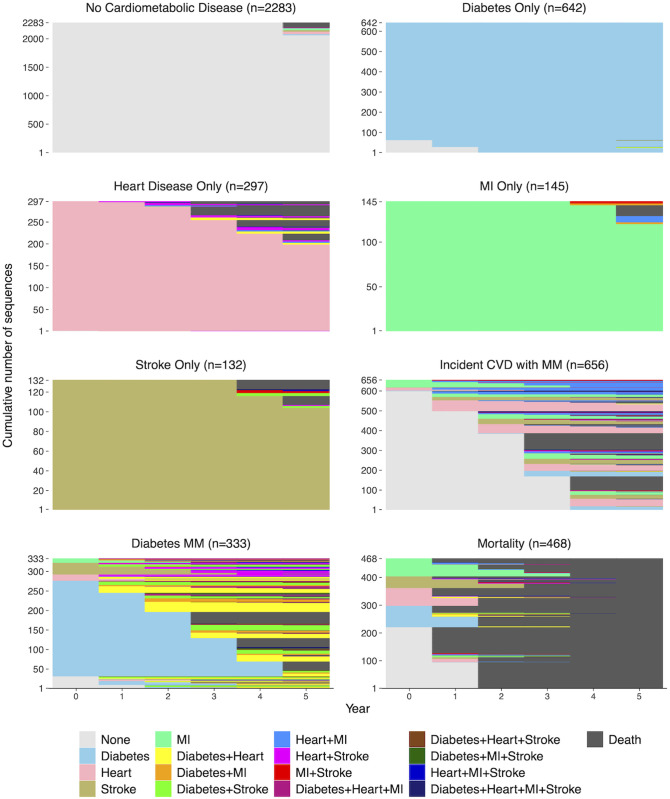
Cardiometabolic disease sequences by cluster. This sequence index plot displays the constituent disease sequences within each of the clusters determined by cluster analysis. Sequences are represented in state sequence format.

Evaluation of fit indices and visual examination of the results of cluster analyses ranging from 2–12 clusters indicated that an eight-cluster solution was optimal. These clusters are depicted using a sequence index plot (Fig 3), which depicts the composition of the identified clusters by displaying each of the constituent sequences in STS format. The largest cluster (N = 2283) almost exclusively consisted of persons who did not develop any of the assessed cardiometabolic conditions across the study period and is thus labeled “*No Cardiometabolic Disease*”. The next four clusters primarily consisted of persons with single cardiometabolic conditions who did not progress to CMM (respectively labeled “*Diabetes Only*” (N=642), *Heart Disease Only*” (N = 297), “*MI Only*” (N = 145), and “*Stroke Only*” (N = 132)). The sixth cluster (N = 656), labeled “*Incident CVD with Multimorbidity*” was primarily composed of persons with no cardiometabolic conditions at baseline (N=601, 91.6%), the majority of whom develop incident cardiovascular disease and/or CMM during the observation period (N = 477, 72.7%). In contrast, the seventh cluster (N = 333), labeled “*Diabetes Multimorbidity*” was dominated by persons with diabetes who developed incident CMM (N = 226, 67.9%) and secondarily by persons with diabetes at baseline who died during the observation period (N = 74, 22.2%). Finally, the eighth cluster (N = 468) was characterized by mortality early in the observation period with minimal CMM development during the observation period. Additional plots depicting the time spent in each state by cluster and the cross-sectional state distribution over time (chronogram) in each cluster are provided in S2 and S3 Figs, respectively.

The 20 most common sequences in the *Incident CVD with Multimorbidity* cluster and the *Diabetes MM* cluster are depicted in sequence index plots using DSS format in S4 Fig. The most common sequences in the *Incident CVD with Multimorbidity* cluster were single transitions from no cardiometabolic disease to, in descending order of frequency, heart disease (19.2%), stroke (9.9%), myocardial infarction (7.8%), and diabetes (6.2%). The three most common multimorbidity sequences in this cluster were baseline MI with subsequent development of heart disease (4.7%), concurrent report of MI and heart disease with no prior cardiometabolic disease (3.0%), and baseline heart disease followed by MI (2.3%). In contrast, 97.0% of the persons in the *Diabetes Multimorbidity* cluster reported diabetes alone at their baseline wave, with the most common cardiometabolic sequences being baseline diabetes with subsequent development of heart disease (19.2%), stroke (10.2%), and MI (6.0%).

### Profile of cardiometabolic disease sequence cluster members

Sociodemographic and health characteristics for each sequence cluster are presented in Table 2. There was a disproportionate number of non-Hispanic Black and Hispanic respondents in both the *Diabetes Only* and the *Diabetes Multimorbidity* clusters, whereas non-Hispanic White respondents were overrepresented in the *Heart Disease Only* cluster. Average cognitive score was highest in the *No Cardiometabolic Diseas*e cluster (Mean = 19.6; SD = 4.3) and lowest in the *Diabetes Multimorbidity* (Mean = 17.4; SD = 4.5) and *Mortality* (Mean = 15.6; SD = 4.2) clusters. These two clusters also had the lowest household incomes, greatest proportion of respondents with less than a high school education, and highest average ADL/IADL limitations.

**Table 2:** Demographic and health characteristics of the cardiometabolic sequence clusters.

Characteristic	No CVD/Met Disease N = 2,283	Diabetes Only N = 642	Heart Disease Only N = 297	MI Only N = 145	Stroke Only N = 132	Incident CVD with MM N = 656	Diabetes MM N = 333	Mortality N = 468
**Age** ^*1*^	74.9 (7.0)	74.1 (6.3)	77.7 (7.6)	77.2 (7.1)	76.9 (7.0)	77.6 (7.6)	76.6 (7.2)	82.2 (7.7)
**Female** ^*2*^	1418 (62.1)	360 (56.1)	173 (58.2)	59 (40.7)	74 (56.1)	369 (56.3)	194 (58.3)	259 (55.3)
**Race/Ethnicity** ^*2*^								
*Non-Hispanic White*	1813 (79.4)	371 (57.8)	256 (86.2)	118 (81.4)	97 (73.5)	509 (77.6)	200 (60.1)	354 (75.6)
*Non-Hispanic Black*	370 (16.2)	214 (33.3)	32 (10.8)	25 (17.2)	32 (24.2)	118 (18.0)	103 (30.9)	94 (20.1)
*Hispanic*	100 (4.4)	57 (8.9)	9 (3.0)	2 (1.4)	3 (2.3)	29 (4.4)	30 (9.0)	20 (4.3)
**Born in US** ^*2*^	2115 (92.6)	580 (90.3)	274 (92.3)	140 (96.6)	125 (94.7)	603 (91.9)	300 (90.1)	434 (92.7)
**Education** ^*2*^								
*Less than high school*	346 (15.2)	142 (22.1)	47 (15.8)	30 (20.7)	35 (26.5)	145 (22.1)	88 (26.4)	149 (31.8)
*High school graduate*	575 (25.2)	196 (30.5)	60 (20.2)	41 (28.3)	42 (31.8)	186 (28.4)	96 (28.8)	129 (27.6)
*Some college*	494 (21.6)	130 (20.2)	72 (24.2)	37 (25.5)	27 (20.5)	116 (17.7)	76 (22.8)	109 (23.3)
*College graduate*	868 (38.0)	174 (27.1)	118 (39.7)	37 (25.5)	28 (21.2)	209 (31.9)	73 (21.9)	81 (17.3)
**Partnered** ^*2*^	1297 (56.8)	324 (50.5)	158 (53.2)	77 (53.1)	67 (50.8)	353 (53.8)	157 (47.3)	183 (39.1)
**Household Income** (in thousands)^*1*^	67.0 (174.0)	44.5 (56.0)	57.2 (85.04)	43.9 (58.7)	46.0 (59.5)	57.6 (210.3)	45.8 (127.8)	30.2 (32.4)
**Non-Cardiometabolic Conditions** ^*1*^	1.58 (1.08)	1.84 (1.02)	2.11 (1.14)	1.78 (1.09)	2.01 (1.04)	1.79 (1.07)	2.10 (0.99)	2.06 (1.15)
**Body Mass Index** ^*1*^	27.4 (5.2)	30.3 (6.1)	27.7 (5.6)	27.2 (4.4)	28.4 (6.4)	27.4 (5.4)	30.4 (6.5)	26.4 (6.2)
**Self-Rated Health** ^*2*^								
*Excellent*	569 (24.9)	34 (5.3)	27 (9.1)	19 (13.2)	9 (6.8)	105 (16.0)	10 (3.0)	28 (6.0)
*Very good*	896 (39.3)	160 (24.9)	79 (26.6)	48 (33.3)	38 (28.8)	224 (34.1)	76 (22.8)	96 (20.5)
*Good*	590 (25.9)	295 (46.0)	113 (38.0)	51 (35.4)	54 (40.9)	198 (30.2)	123 (36.9)	160 (34.2)
*Fair*	199 (8.7)	136 (21.2)	57 (19.2)	21 (14.6)	20 (15.2)	101 (15.4)	95 (28.5)	128 (27.4)
*Poor*	28 (1.2)	17 (2.6)	21 (7.1)	5 (3.5)	11 (8.3)	28 (4.3)	29 (8.7)	56 (12.0)
**Number of ADL/IADL Limitations** ^*1*^	0.8 (1.7)	1.3 (2.1)	1.6 (2.5)	1.1 (1.8)	1.9 (2.7)	1.3 (2.4)	2.0 (2.6)	2.7 (3.1)
**Cognitive Score** ^*1*^	19.6 (4.3)	18.6 (4.2)	19.0 (4.3)	17.9 (4.2)	18.3 (4.2)	18.4 (4.7)	17.4 (4.5)	15.6 (4.2)

^*1*^Mean (SD); ^*2*^N (%); ADL = Activities of Daily Living; IADL = Instrumental Activities of Daily Living.

The results of a multinomial logistic regression model of the association of cluster membership to baseline sociodemographic predictors and BMI are presented in Table 3. The *No Cardiometabolic Disease* cluster served as the reference group. With the exception of the *Diabetes Only* cluster, advanced age was consistently associated with significantly greater odds of being in all of the cardiometabolic disease clusters after adjusting for covariates. Females had significantly lower odds than males of being in all of the cardiometabolic disease clusters except the *Heart Disease Only* cluster.

**Table 3:** Multinomial logistic regression of cardiometabolic sequence cluster membership.

Characteristic	Diabetes Only	Heart Disease Only	MI Only	Stroke Only	Incident CVD with MM	Diabetes MM	Mortality
**Age**	0.99 (0.98, 1.01)	1.06*** (1.04, 1.08)	1.04*** (1.02, 1.07)	1.04** (1.01, 1.07)	1.05*** (1.04, 1.07)	1.05*** (1.03, 1.07)	1.13*** (1.11, 1.14)
**Female**	0.66*** (0.54, 0.81)	0.77 (0.59, 1.01)	0.31*** (0.21, 0.45)	0.65* (0.44, 0.96)	0.71*** (0.59, 0.87)	0.64*** (0.50, 0.84)	0.47*** (0.37, 0.60)
**Race/Ethnicity** *(Ref = Non-Hispanic White)*							
* Non-Hispanic Black*	2.29*** (1.83, 2.85)	0.60* (0.40, 0.89)	0.81 (0.50, 1.30)	1.29 (0.82, 2.02)	0.99 (0.77, 1.27)	2.07*** (1.55, 2.76)	1.04 (0.78, 1.39)
* Hispanic*	2.25*** (1.47, 3.47)	0.62 (0.30, 1.27)	0.20* (0.05, 0.83)	0.4 (0.11, 1.46)	0.84 (0.54, 1.32)	1.98** (1.23, 3.19)	0.54* (0.31, 0.95)
**Coupled**	0.92 (0.74, 1.14)	1.04 (0.78, 1.40)	1.01 (0.67, 1.52)	1.03 (0.67, 1.57)	1.20 (0.96, 1.48)	1.01 (0.76, 1.35)	1.07 (0.82, 1.39)
**Education** *(Ref = High school graduate)*							
* Less than high school*	0.87 (0.66, 1.15)	1.22 (0.80, 1.87)	1.03 (0.62, 1.73)	1.28 (0.78, 2.09)	1.07 (0.81, 1.40)	1.06 (0.75, 1.51)	1.44* (1.07, 1.95)
* Some college*	0.82 (0.63, 1.06)	1.52* (1.05, 2.20)	1.21 (0.76, 1.94)	0.81 (0.49, 1.34)	0.8 (0.61, 1.04)	1.05 (0.75, 1.48)	1.21 (0.89, 1.64)
* College graduate*	0.71** (0.55, 0.91)	1.58* (1.10, 2.25)	0.71 (0.43, 1.18)	0.50* (0.29, 0.84)	0.88 (0.68, 1.12)	0.69* (0.49, 0.98)	0.68* (0.49, 0.95)
**Household Income** *(Ref = Q4)*							
* Q3*	1.23 (0.95, 1.59)	1.26 (0.91, 1.75)	1.2 (0.72, 1.98)	1.26 (0.75, 2.10)	1.07 (0.84, 1.38)	1.33 (0.94, 1.90)	1.97*** (1.37, 2.85)
* Q2*	1.38* (1.03, 1.86)	1.38 (0.93, 2.05)	1.91* (1.10, 3.32)	1.19 (0.66, 2.15)	1.34* (1.01, 1.79)	1.32 (0.88, 1.97)	3.19*** (2.17, 4.68)
* Q1*	1.49* (1.06, 2.10)	1.61* (1.02, 2.55)	2.91*** (1.59, 5.32)	1.63 (0.85, 3.12)	1.76*** (1.27, 2.44)	1.82** (1.17, 2.82)	4.15*** (2.73, 6.31)
**BMI Category** *(Ref=<25)*							
* 25-29.9*	1.67*** (1.30, 2.15)	0.88 (0.66, 1.18)	0.95 (0.63, 1.44)	0.92 (0.60, 1.41)	1.01 (0.82, 1.25)	1.29 (0.92, 1.79)	0.61*** (0.49, 0.82)
* 30+*	3.08*** (2.39, 3.97)	1.38* (1.01, 1.91)	1.21 (0.77, 1.92)	1.22 (0.77, 1.94)	1.23 (0.97, 1.56)	3.23*** (2.34, 4.48)	0.89 (0.67, 1.18)

*p < 0.05; **p < 0.01; ***p < 0.001; MI = Myocardial infarction; CVD = Cardiovascular disease; MM = Multimorbidity;  BMI =   Body mass index.

Non-Hispanic Black respondents had significantly greater odds than non-Hispanic Whites of being in the *Diabetes Only* cluster (aOR=2.29; 95% CI = 1.83,2.85) and the *Diabetes Multimorbidity* cluster (aOR=2.07; 95% CI = 1.55,2.76), but significantly lower odds of being in the *Heart Disease Only* cluster (aOR=0.60; 95% CI = 0.40,0.89). Similarly, Hispanic respondents had significantly greater odds than non-Hispanic Whites of being in the *Diabetes Only* cluster (aOR=2.25; 95% CI = 1.47,3.47) and the *Diabetes Multimorbidity* cluster (aOR=1.98; 95% CI = 1.23,3.19), but significantly lower odds of being in the *MI Only* cluster (aOR=0.20; 95% CI = 0.05,0.83) and the *Mortality cluster* (aOR=0.20; 95% CI = 0.05,0.83). In addition, both educational attainment and household income were significantly associated with cardiometabolic sequence cluster in adjusted models, with higher levels of both income and educational attainment being generally associated with lower odds of membership in a cardiometabolic disease cluster and lower odds of being in the *Mortality* cluster.

Baseline BMI was significantly associated with cluster membership in the *Diabetes Only*, *Heart Disease Only*, *Diabetes Multimorbidity*, and *Mortality* clusters. Relative to healthy weight persons [18.5 ≤ BMI < 30], both overweight [25 ≤ BMI < 30] (aOR=1.67; 95% CI = 1.30,2.15) and obese [BMI ≥ 30] (aOR=3.08; 95% CI = 2.39,3.97) persons had greater odds of *Diabetes Only* cluster membership. However, only the obese category was associated with significantly greater odds of membership in the *Diabetes Multimorbidity* cluster (aOR=3.23; 95% CI = 2.34,4.48) and obesity was also associated with increased odds of membership in the *Heart Disease Only* cluster (aOR=1.38; 95% CI = 1.01,1.91). Finally, compared to healthy weight persons, overweight persons had significantly lower odds of being in the *Mortality* cluster (aOR=0.61; 95% CI = 0.49,0.82) in covariate adjusted models. There was not a significant difference in the odds of Mortality cluster membership among obese persons compared to healthy weight persons.

### Dementia outcomes post multimorbidity onset by cluster

**Fig 4:**
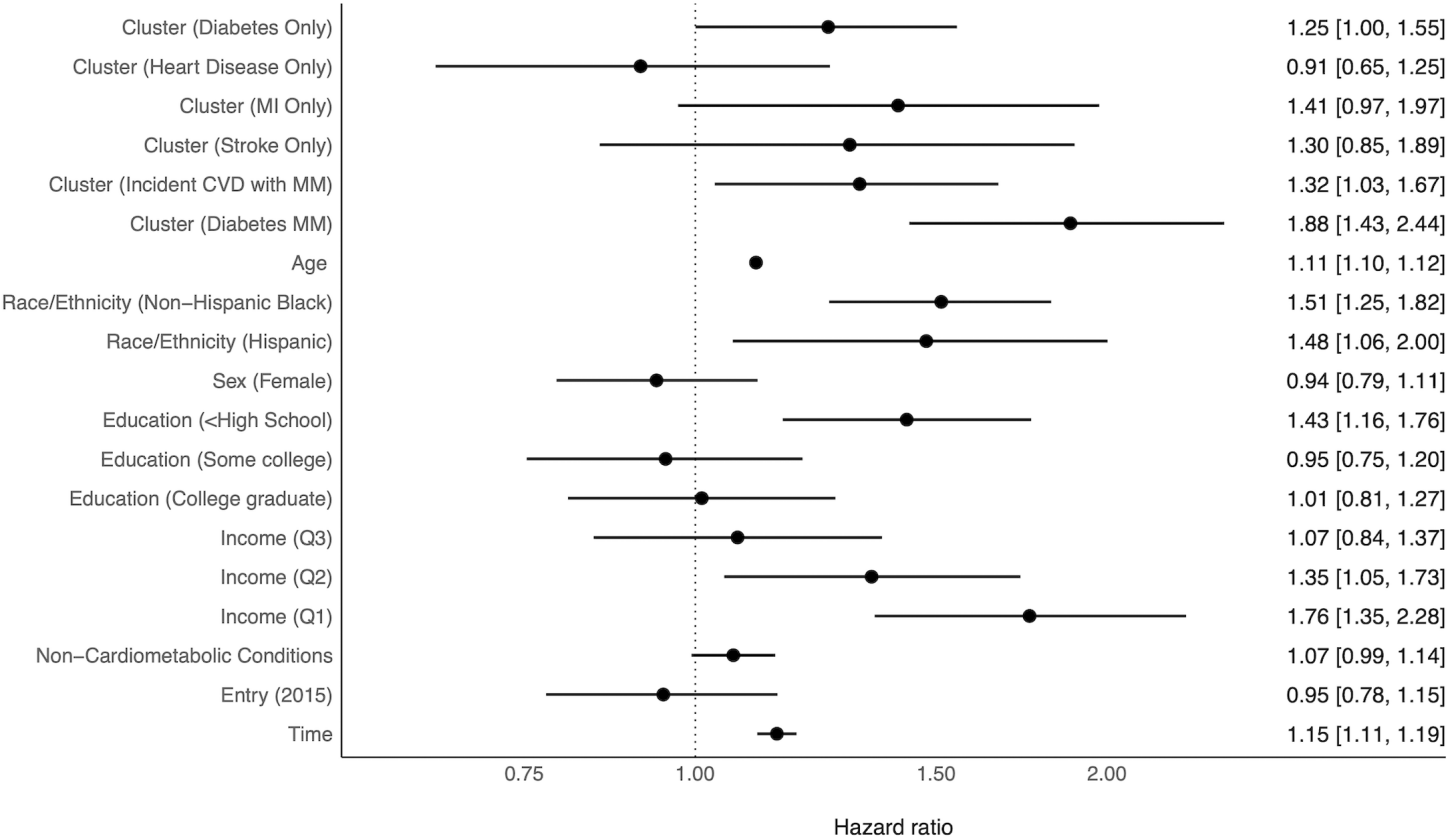
Adjusted hazard ratios and 95% confidence intervals of dementia onset associated with cardiometabolic sequence cluster membership. Models adjusted for baseline demographic characteristics and time varying count of non-cardiometabolic conditions (estimates also shown).

Of the participants who were included in the analysis of dementia risk post multimorbidity cluster ascertainment, 19.1 % (N=761) developed dementia by the end of the study period. The results of the fully-adjusted discrete time survival analysis of the association of dementia onset with cardiometabolic sequence cluster are presented in Fig 4 and in tabular format in Supplemental Table 2. Note that the mortality cluster is not included in this model as all members of this cluster expired prior to dementia ascertainment. We observed no violation of the proportional odds assumption in our testing. Among the identified cardiometabolic sequence clusters, persons in the *Diabetes Only* (aHR = 1.25; 95% CI = 1.00,1.55), *Incident CVD with Multimorbidity* (aHR = 1.32; 95% CI = 1.04,1.67), and the *Diabetes Multimorbidity* (aHR = 1.88; 95% CI = 1.44,2.44) clusters had significantly increased risk of dementia compared with the *No Cardiometabolic Disease* cluster in the covariate-adjusted model.

Both non-Hispanic Black (aHR = 1.51; 95% CI = 1.25,1.81) and Hispanic (aHR = 1.49; 95% CI = 1.07,2.02) respondents had significantly greater hazard of dementia relative to non-Hispanic White respondents in fully adjusted models. Compared to the highest income quartile, persons in the bottom two quartiles had significantly greater dementia risk, with increases in hazard of 32% (aHR = 1.32; 95% CI = 1.02,1.71) and 71% (aHR = 1.71; 95% CI = 1.29,2.26) for quartiles three and four, respectively. Persons who did not graduate high school were significantly more likely (aHR = 1.43; 95% CI = 1.16,1.76) to develop dementia compared to high school graduates, although we did not observe significant differences in the hazard of dementia among college-educated persons relative to high school graduates. We observed no significant association between time to dementia and dementia and sex, coupled status, or the count of non-cardiometabolic chronic conditions in fully adjusted models.

## Discussion

This study assessed cardiometabolic multimorbidity patterns and sequencing of occurrence of constituent CMM diseases among a nationally-representative sample of traditional Medicare beneficiaries. The most common sequence observed among the sample was that defined by the absence of any cardiometabolic disease across the observation period. On average, respondents without cardiometabolic disease during the study period were younger, had higher levels of income and educational attainment, had lower average BMI, and had fewer non-cardiometabolic chronic conditions than respondents with cardiometabolic disease. Roughly one in ten persons in the sample developed CMM within 5 years. This aligns with previously reported estimates of CMM in both national and international cohorts of older adults and supports the recognition that CMM is an area of growing concern for older populations [40,41]. We identified distinct cardiometabolic disease clusters characterized by their patterns of cardiometabolic disease accumulation over the five-year follow-up period. Of these, two were primarily multimorbidity clusters, one predominately consisting of baseline diabetes followed by the development of additional cardiovascular conditions (*Diabetes Multimorbidity*), and one consisting of persons without any cardiometabolic disease at baseline who reported developing one or more conditions during the follow-up period (*Incident CVD with Multimorbidity*).

We observed racial/ethnic differences in the prevalence of specific cardiometabolic conditions, the temporal pattering of cardiometabolic disease accumulation, and the risk of CMM. Non-Hispanic Black and Hispanic older adults were more significantly more likely than non-Hispanic White older adults to both have diabetes at baseline and to develop a cardiovascular condition combined with diabetes. These findings are consistent with the markedly increased prevalence of diabetes observed among these racial/ethnic minority populations in the US [42]. In contrast, compared with non-Hispanic White participants, non-Hispanic Black and Hispanic older adults had reduced odds of membership in the Heart *Disease Only* and *MI Only* clusters, respectively, and there were no differences between racial/ethnic groups in the likelihood of being in the *Incident CVD with Multimorbidity* cluster. Previous studies have reported that the incidence of coronary heart disease is higher among non-Hispanic White compared to non-Hispanic Black persons, although the incidence of stroke is higher among non-Hispanic Black persons [43]. Similarly, the lower reduced odds of being in the *MI Only* cluster among Hispanic older adults is consistent with recent estimates of myocardial infarction incidence by race/ethnicity [44], although the magnitude of the difference is larger than observed in previous studies.

Both lower household income and greater educational attainment were significantly associated with CMM development during the observation period. Older adults in the lowest income quartile had increased odds of being in both the Diabetes Multimorbidity and the Incident CVD with Multimorbidity clusters, and college graduates having lower odds of membership in the Diabetes Only, Stroke Only, Diabetes Multimorbidity and Mortality clusters compared to high school graduates, although college graduates had higher odds of membership in the Heart Disease Only cluster. These findings are broadly consistent with prior research on social determinants of cardiometabolic disease [39,45]. Increased BMI was associated with cluster membership in varying ways depending on BMI category. Persons who were obese at baseline had, compared with persons with healthy weight, a greater than three-fold increase in the odds of membership in the Diabetes Only and Diabetes Multimorbidity clusters, as well as increased likelihood of being in the Heart Disease Only cluster. In contrast, while overweight persons had significantly greater odds than persons with BMI < 25 of being in the Diabetes Only cluster, there was no difference in the odds of being in either of the multimorbidity clusters and overweight persons had significantly reduced odds of membership in the Mortality cluster. The potentially protective effect of BMI in the overweight range on mortality among older adults has been documented in a number of previous studies [46–48]. Finally, we observed females to have significantly reduced odds of being in nearly all of cardiometabolic disease clusters as well as the Mortality cluster relative to males.

The results of the discrete time survival models revealed significantly increased risk of dementia onset among participants in the *Diabetes Only*, *Diabetes Multimorbidity*, and *Incident CVD with Multimorbidity* clusters. Of these, the magnitude of the increase in the hazard of dementia was greatest for those in the *Diabetes Multimorbidity* group, whose dementia hazard was nearly twice that of participants in the *No Cardiometabolic Disease* cluster. These findings support the growing body of evidence that CMM may be a substantial risk factor for the development of cognitive impairment and dementia.

Our results support and extend those reported in several recent studies. Jin et al. [19] reported a significant and dose-dependent association between increased number of cardiometabolic conditions and declining cognitive function among a pooled cohort drawn from the Health and Retirement Study and three harmonized international cohorts. Dove et al. [21] report a roughly two-fold increase in the hazard of dementia, as ascertained by diagnostic codes, among older adults with CMM in the Swedish Twin Registry. Zhang et al. [22] observed an increase in the risk of motoric cognitive risk (MCR), defined by cognitive complaints in the presence of slow gait speed, among participants with both single and multiple cardiometabolic conditions (diabetes, heart disease, stroke). Our findings differ in that we did not observe an association between dementia hazard and any of the assessed cardiovascular conditions in isolation. However, our outcome of “probable dementia” is based on a conservative cutoff in the cognitive score and is indicative of advanced cognitive impairment relative to the MCR outcome, which captures earlier changes in cognitive function.

Our finding that both non-Hispanic Black and Hispanic older adults had greater odds of diabetes multimorbidity, combined with the substantial increase in risk of cognitive decline among persons with CMM, suggests that CMM may play a role in observed racial/ethnic disparities in dementia. Thus, CVD prevention and risk reduction among minoritized older adults with uncomplicated diabetes may emerge as an effective means of slowing or preventing the onset of cognitive decline among these groups. However, we did observe increased risk of dementia onset among both non-Hispanic Black and Hispanic respondents in models adjusted for cardiometabolic sequence cluster, suggesting that the contribution of cardiometabolic disease does not fully explain observed racial/ethnic differences in dementia risk and that addressing this requires multifactorial approaches.

Several limitations of this study should be noted. First, the inclusion of cases with missing data in sequence analysis is currently limited to representing the state of missingness as a discrete state. While we did include attrition due to mortality as a discrete state in the analysis, thus retaining those participants who died during the 5-year period during which the CMM sequences were observed, the inclusion of an additional state to represent non-mortality attrition resulted in a marked increase in the number of unique sequences and a lack of convergence in cluster solutions. Thus, the described CMM sequences represent those observed among the cohort who had either complete data for five-years post-baseline or who died during the follow-up period and may be subject to bias as a result. However, any such bias would almost certainly result in an underestimation of the proportion of the sample who developed CMM, and thus our reported estimates should be viewed as conservative. Second, the presence of cardiometabolic conditions are self-reported and may be subject to under-reporting or misreporting. However, multiple studies have shown adequate concordance between patient self-reports and objectively ascertained diagnoses [49,50]. Third, there is substantial heterogeneity in the Hispanic ethnicity category in regard to country of origin and/or ancestry which is associated with differential health outcomes between these subgroups [51]. Furthermore, there was insufficient numbers of other racial and ethnic groups present in the NHATS cohort to include in our analyses. Future work should leverage larger cohorts or administrative databases to describe and compare CMM sequences between Hispanic subgroups and among understudied racial/ethnic groups given the high prevalence of cardiometabolic disease present among these populations.

In conclusion, this study used a novel approach in the multimorbidity literature, sequence analysis, to describe a typology of cardiometabolic disease sequences based on the timing, ordering, and combining of conditions over time and to identify the demographic predictors and cognitive outcomes associated with the different clusters within that observed typology. Our results show that the development of cardiometabolic multimorbidity is associated with increased risk of dementia in adults aged 65 years and older, such that persons with diabetes who accumulated additional cardiovascular disease during the follow-up period had nearly a two-fold risk of subsequent dementia compared with those without cardiometabolic disease. Minoritized, low-income, and obese older adults were more likely to be in the highest risk CMM group. These finding support the growing evidence of cardiometabolic multimorbidity as an important risk factor for cognitive decline and underscore the urgent need to develop targeted public health approaches to prevent diabetes and related cardiovascular sequela in order to address racial/ethnic and socioeconomic disparities in dementia. This study demonstrates the utility of sequence analysis as a methodological approach to the study of multimorbidity development and progression and provides important evidence regarding the impact of CMM on cognitive function among older adults.

## Supporting information

S1 Fig Sequence index plot of observed cardiometabolic disease sequences in the full analytic sample. This plot displays all observed cardiometabolic disease sequences in the sample over the five-year observation period in state sequence format, in which the disease state at each observed time point is represented.
(TIF)

S2 Fig Mean time plot by cluster. This plot depicts the mean number of survey waves (including baseline) that respondents spent in each cardiometabolic disease state stratified by cluster.
(TIF)

S3 Fig Chronogram of cardiometabolic disease sequences by cluster. This chronogram depicts the cross-sectional distribution of cardiometabolic disease states at each time point within each cluster. The cumulative proportion is represented on the y-axis.
(TIF)

S4 Fig Relative frequency of 20 most common cardiometabolic disease sequences in multimorbidity clusters. This sequence frequency plot displays the twenty most commonly occurring cardiometabolic disease sequences in reduced (DSS) format among persons in the “Incident CVD with Multimorbidity” cluster and the “Diabetes Multimorbidity” cluster. The proportion of respondents with each sequence (relative to the total sample in that cluster) is displayed on the y-axis.
(TIF)

S5 Fig Flowchart of analytic sample construction. This flowchart depicts the construction of the analytic sample through application of study inclusion and exclusion criteria to the full NHATS cohort.
(TIF)

S1 File R code used to conduct analysis. (RTF)

S1 Table Twenty most frequently occurring cardiometabolic disease sequences in distinctive successive state (DSS) format. (DOCX)

S2 Table Discrete time survival analysis of time to dementia onset by cardiometabolic sequence cluster. (DOCX)
