# Supplementary figures and images for: Longitudinal sequencing of cardiometabolic multimorbidity among older adults and association with subsequent dementia onset
Source: PLoS One. 2025 Jul 10;20(7):e0326309. doi: 10.1371/journal.pone.0326309 (PMC12244708; doi:10.1371/journal.pone.0326309)

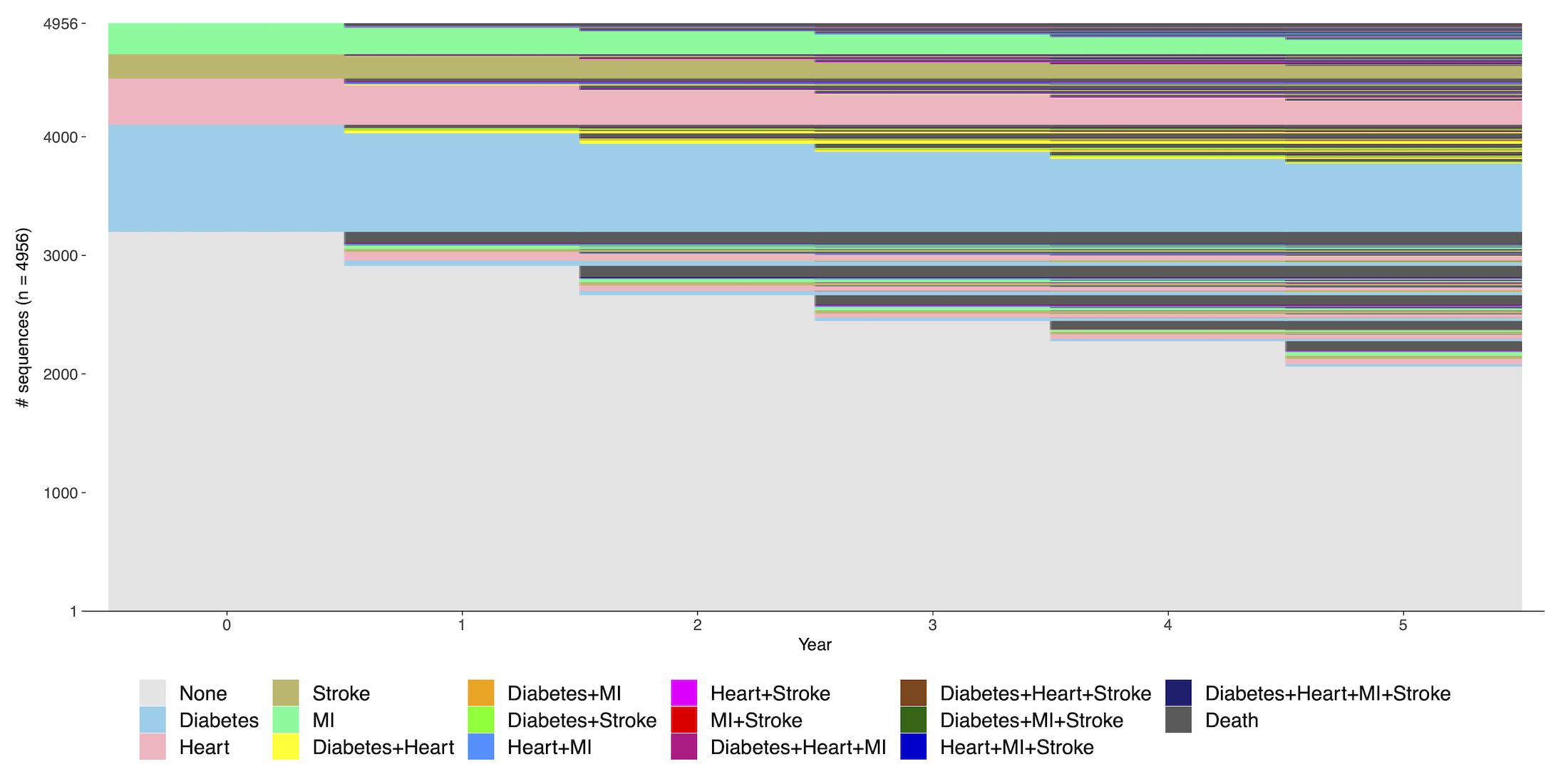

Supplement: S1 Fig — This plot displays all observed cardiometabolic disease sequences in the sample over the five-year observation period in state sequence format, in which the disease state at each observed time point is represented. (TIF) [file pone.0326309.s001.tif]

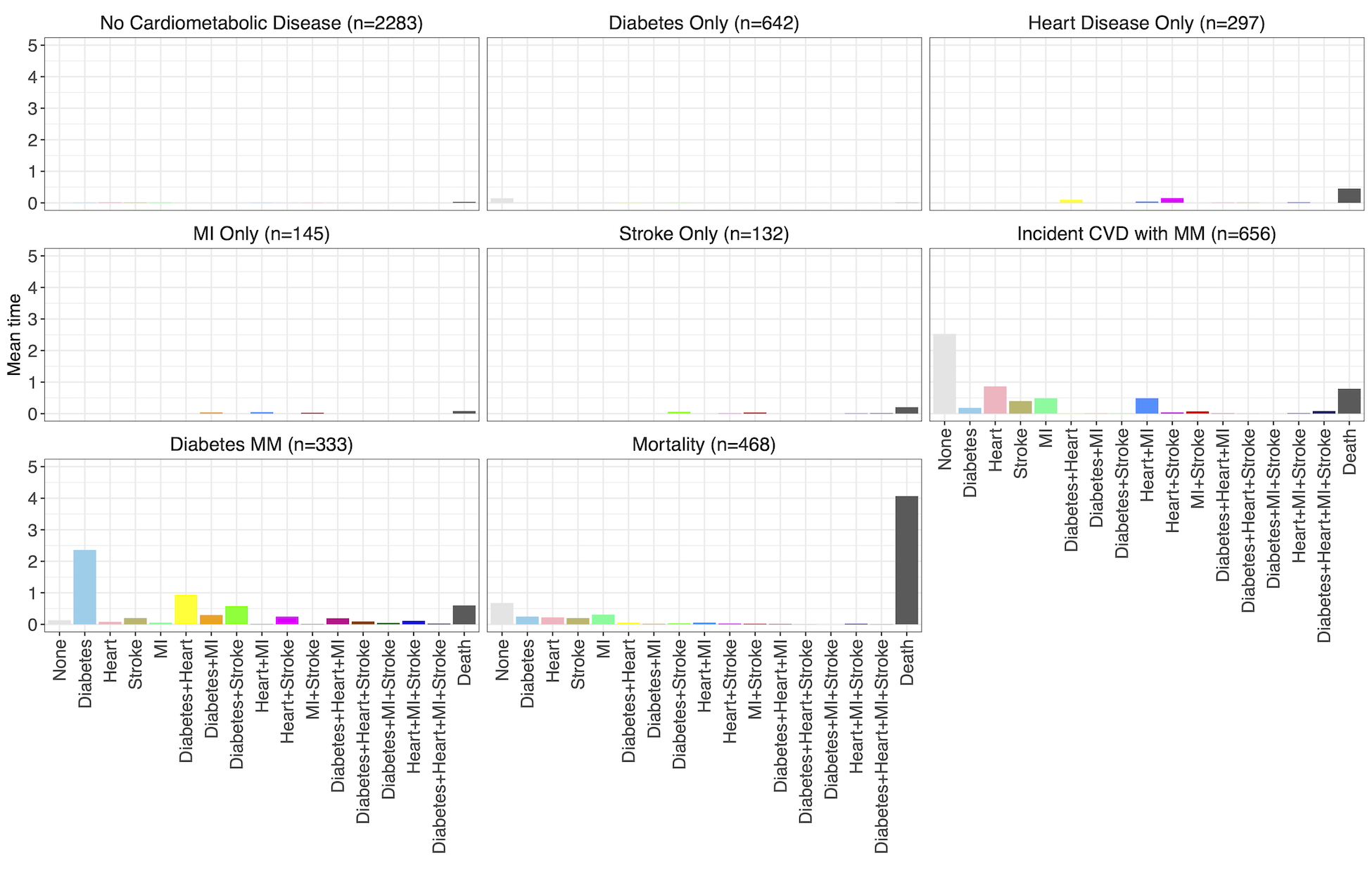

Supplement: S2 Fig — This plot depicts the mean number of survey waves (including baseline) that respondents spent in each cardiometabolic disease state stratified by cluster. (TIF) [file pone.0326309.s002.tif]

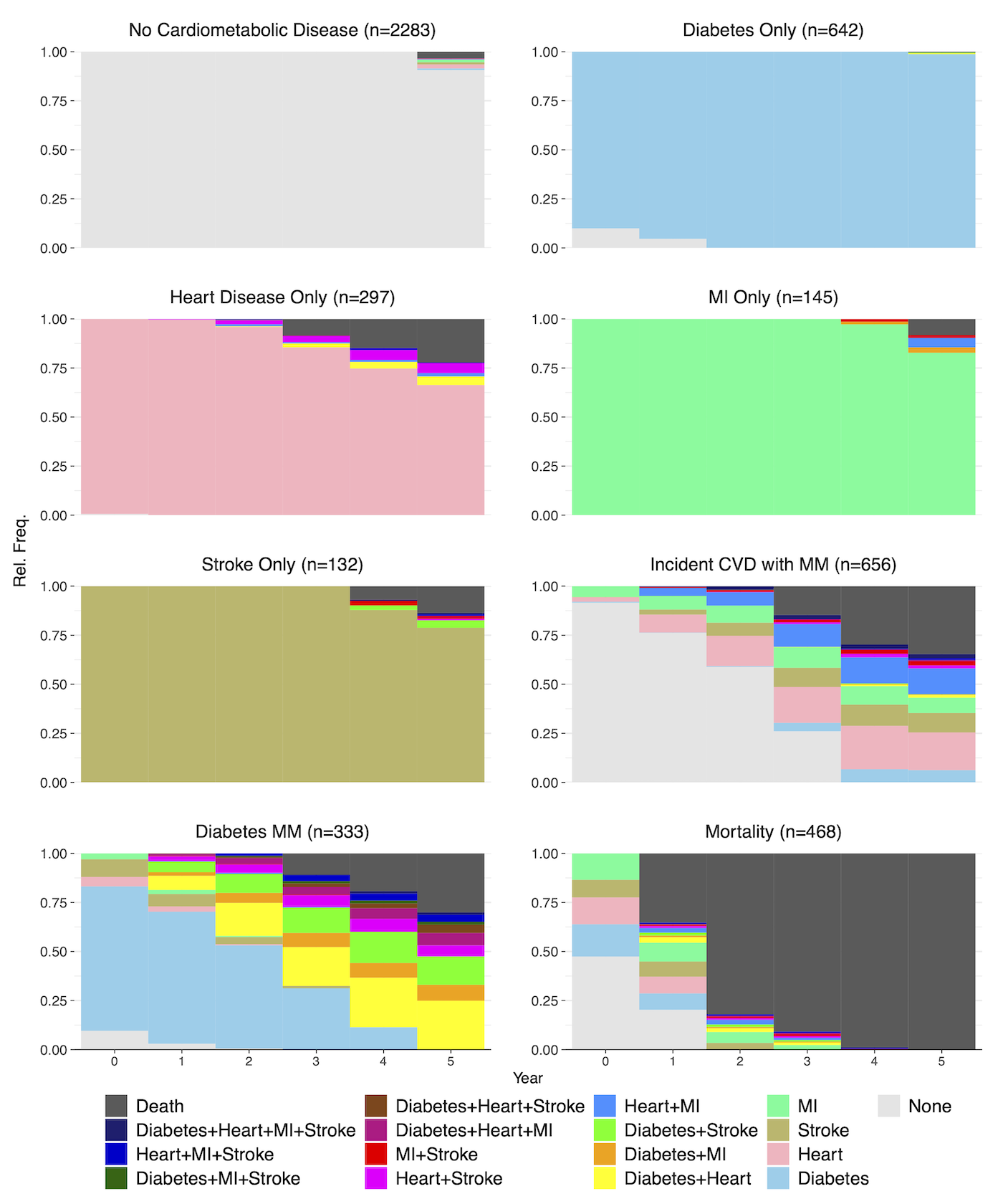

Supplement: S3 Fig — This chronogram depicts the cross-sectional distribution of cardiometabolic disease states at each time point within each cluster. The cumulative proportion is represented on the y-axis. (TIF) [file pone.0326309.s003.tif]

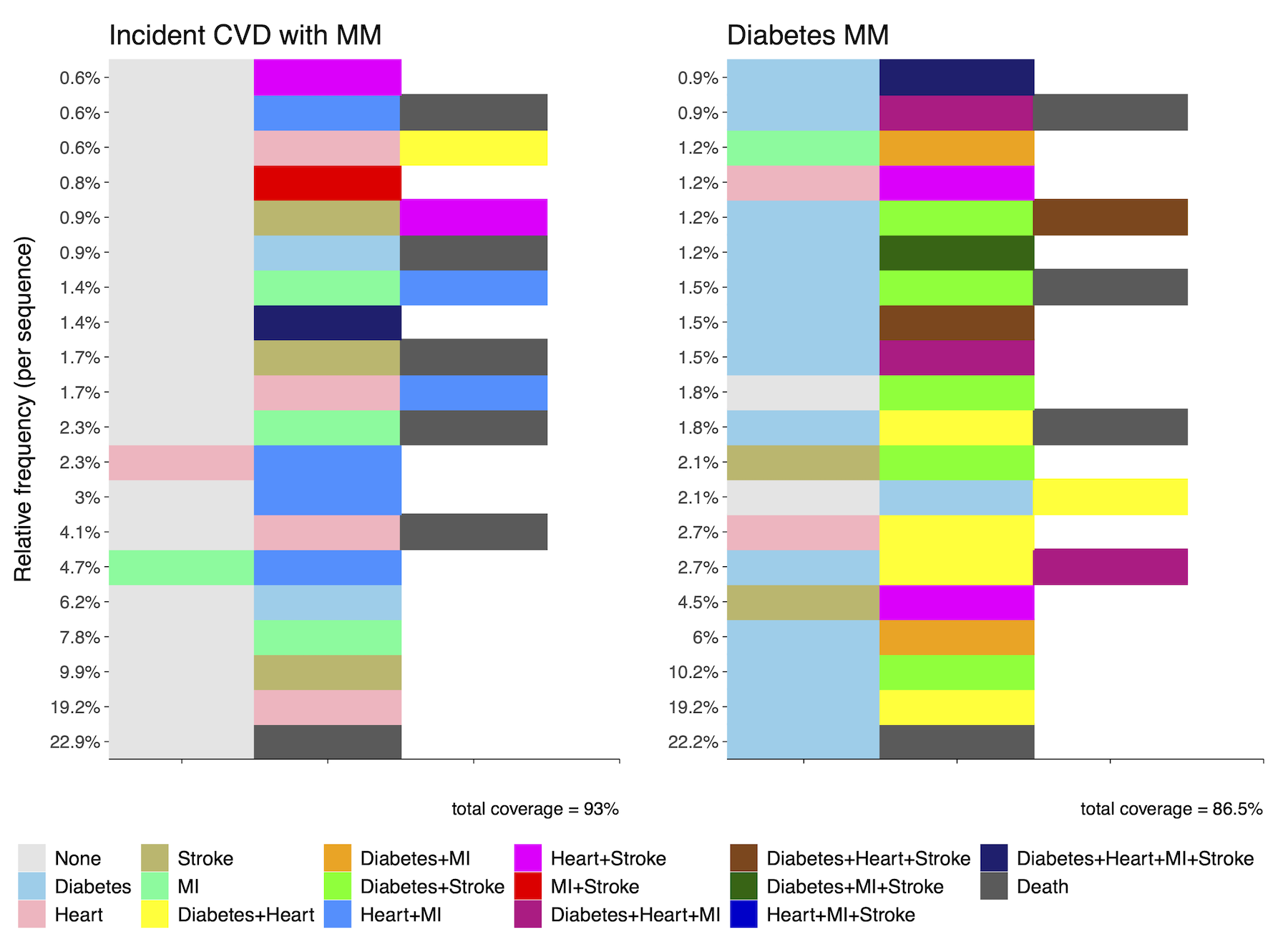

Supplement: S4 Fig — This sequence frequency plot displays the twenty most commonly occurring cardiometabolic disease sequences in reduced (DSS) format among persons in the “Incident CVD with Multimorbidity” cluster and the “Diabetes Multimorbidity” cluster. The proportion of respondents with each sequence (relative to the total sample in that cluster) is displayed on the y-axis. (TIF) [file pone.0326309.s004.tif]

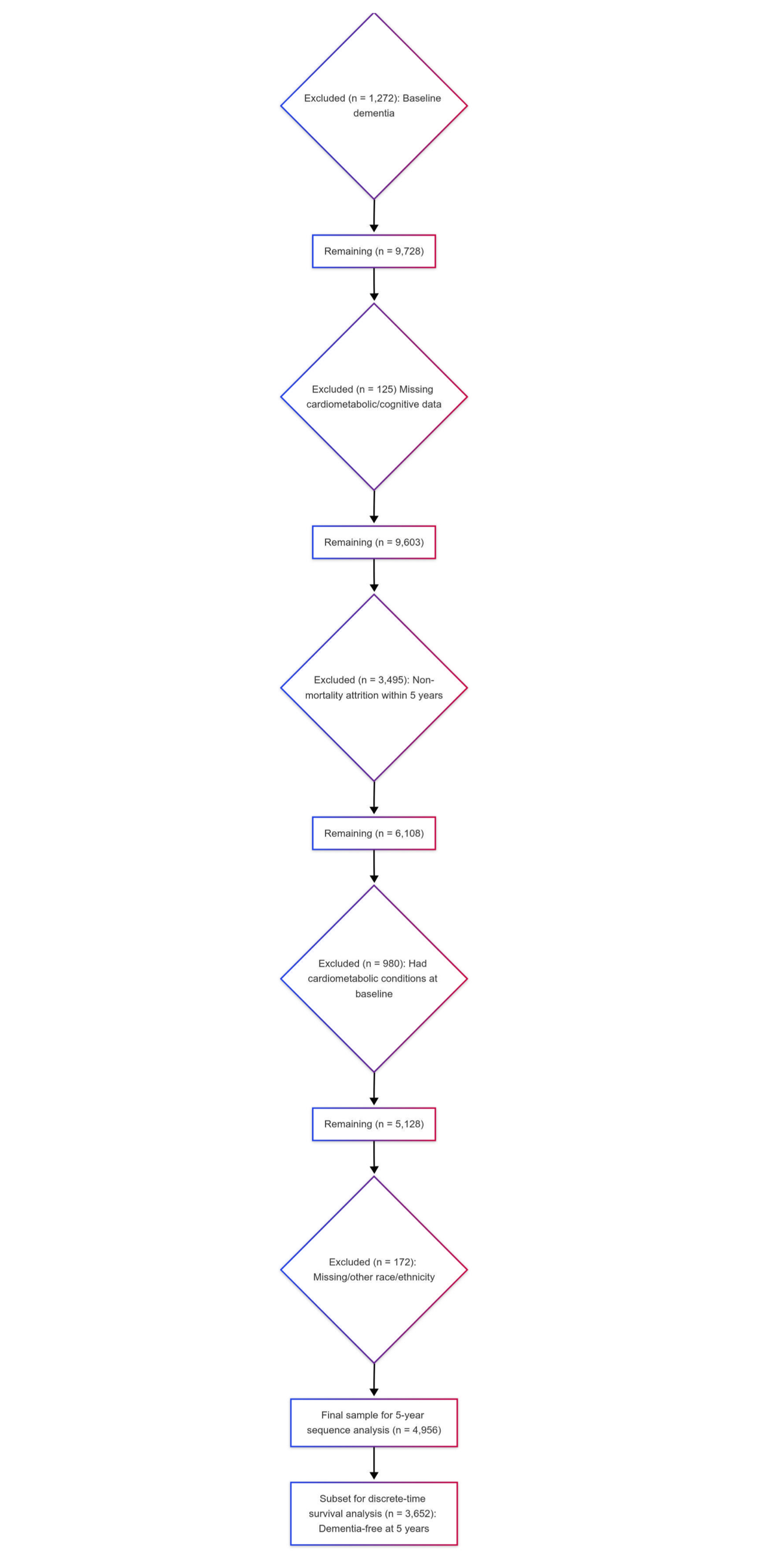

Supplement: S5 Fig — This flowchart depicts the construction of the analytic sample through application of study inclusion and exclusion criteria to the full NHATS cohort. (TIF) [file pone.0326309.s005.tif]
