# Supplementary material for: Longitudinal sequencing of cardiometabolic multimorbidity among older adults and association with subsequent dementia onset
Source: PLoS One. 2025 Jul 10;20(7):e0326309. doi: 10.1371/journal.pone.0326309 (PMC12244708; doi:10.1371/journal.pone.0326309)
Supplement: S1 File — (RTF) [file pone.0326309.s006.rtf]

```{r setup, include=FALSE}knitr::opts_chunk$set(echo = TRUE,options(scipen=999))``````{r, message=F, warning=F}library(dplyr)library(ggplot2)library(forcats)library(janitor)library(kableExtra)library(knitr)library(colorspace)library(TraMineR)library(TraMineRextras)```Read in data```{r}load("nhats_mm7.RData")```Create color pallete using maximally contrasting colors```{r}colors1 <- c("#e5e5e5","#87CEEB", "#FFB6C1", "#BDB76B", "#00FA9A", "#FFFF00", "#FFA500", "#00FF00", "#1E90FF", "#FF00FF", "#FF0000", "#C71585", "#8B4513", "#006400", "#0000CD", "#191970","#5A5A5A")swatchplot(colors1)```Sequence object for mm7```{r}mm7 <- seqdef(nhats_mm7,              var=2:7,              alphabet = c("none","diab","hrt","stk","mi","diab/hrt",                               "diab/mi","diab/stk","hrt/mi","hrt/stk",                               "mi/stk","diab/hrt/mi","diab/hrt/stk","diab/mi/stk",                               "hrt/mi/stk","diab/hrt/mi/stk","death"),              labels = c("None","Diabetes","Heart","Stroke","MI","Diabetes+Heart",                               "Diabetes+MI","Diabetes+Stroke","Heart+MI","Heart+Stroke",                               "MI+Stroke","Diabetes+Heart+MI","Diabetes+Heart+Stroke","Diabetes+MI+Stroke",                               "Heart+MI+Stroke","Diabetes+Heart+MI+Stroke","Death"))attributes(mm7)$cpal <- colors1```Descriptive statistics on time spent in each state--this is the average number of years spent in each state```{r, warning=F}seqmeant(mm7, serr = TRUE) %>%     as_tibble(rownames = "State") %>%     mutate(across(-c(State), round, 2)) %>%     select(-Var) %>%     kbl() %>%     kable_styling(bootstrap_options = c("responsive", "hover", "condensed"),                   full_width = F)```Proportion of total time spent in each state```{r}seqmeant(mm7, prop = TRUE) %>%     as_tibble(rownames = "State") %>%     mutate(across(-c(State), round, 3)) %>%     rename(Proportion=Mean) %>%     kbl() %>%     kable_styling(bootstrap_options = c("responsive", "hover", "condensed"),                   full_width = F)```State distribution by wave```{r}seqstatd(mm7)$Frequencies %>%     as_tibble(rownames = "State") %>%     mutate(across(2:7, round, 3)) %>%     kable(col.names = c("State",1:6)) %>%    add_header_above(c(" ", "State distribution at wave" = 6)) %>%    kable_styling(bootstrap_options = c("responsive", "hover", "condensed"),                   full_width = F)```Mean number of transitions```{r}mean(seqtransn(mm7))```Labels for tables```{r}mm7lab <- c("none","diab","hrt","stk","mi","diab/hrt",                               "diab/mi","diab/stk","hrt/mi","hrt/stk",                               "mi/stk","diab/hrt/mi","diab/hrt/stk","diab/mi/stk",                               "hrt/mi/stk","diab/hrt/mi/stk","death")```Transition rate at T+1 using STS Format. This pools all individual states in the state space, so there are a proportion who transition between the same state at a given T+1. No state for attrition.```{r}#named vector with transition proportionstrate_p <- seqtrate(mm7) #name rows and columnsrownames(trate_p) <- mm7labcolnames(trate_p) <- mm7labtrate_p <- as_tibble(round(trate_p,3),                     rownames = "Origin")trate_p %>%  mutate(Origin = cell_spec(Origin, bold = TRUE, color = "black")) %>%  kable(col.names = c("State at t","none","diab","hrt","stk","mi","diab/hrt",                               "diab/mi","diab/stk","hrt/mi","hrt/stk",                               "mi/stk","diab/hrt/mi","diab/hrt/stk","diab/mi/stk",                               "hrt/mi/stk","diab/hrt/mi/stk","death"), escape = FALSE) %>%  add_header_above(c(" ", "State at t+1" = 17)) %>%  kable_styling(bootstrap_options =                   c("responsive", "hover", "condensed"),                full_width = F)```Plot of transitions--can be grouped```{r}library(ggseqplot)p1 <- ggseqtrplot(mm7, dss=F,labsize = 3)+    theme(axis.text.x = element_text(angle = 90, vjust = 0.5, hjust=1))+    ggtitle("STS sequences")p2 <- ggseqtrplot(mm7, dss=T,labsize = 3)+    theme(axis.text.x = element_text(angle = 90, vjust = 0.5, hjust=1))+    ggtitle("DSS sequences")pdf("sts_transition_plot.pdf", width=7, height = 7)p1dev.off()pdf("dss_transition_plot.pdf", width=7, height = 7)p2dev.off()p2 <- p2 +  theme(axis.text.y = element_blank(),         axis.title.y = element_blank())p1 + p2 &  theme(plot.title = element_text(size = 20,                                  hjust = 0.5))pdf("combined_transition_plot.pdf", width=14, height = 7)p1 + p2 &  theme(plot.title = element_text(size = 20,                                  hjust = 0.5))dev.off()```Frequency of top 20 sequences in STS format```{r}seqtab(mm7, idxs = 1:20)```count up sts sequences```{r}n <- seqtab(mm7, idxs = 1:500)nrow(n)rm(n)``````{r}library(ggseqplot)p <- ggseqiplot(mm7, border=F, sortv="from.start",)+         scale_x_discrete(labels=0:5, "Year")+       theme(axis.text.y = element_text(size=10))+           theme(axis.text.x = element_text(size=10))+    theme(legend.position = "bottom", legend.justification = c("center"),legend.spacing.x = unit(.3, 'cm'))+    guides(fill=guide_legend(ncol=7))+     theme(legend.text=element_text(size=13))ppdf("total_sts_seq.pdf", width=14, height = 7)pdev.off()```Frequency plot of 20 most common sequences in STS format```{r,out.width="90%"}p <- ggseqfplot(mm7, proportional = F, ylabs="share", ranks=1:20,  na.rm=T)+    theme(axis.text.y = element_text(size=9))+    scale_x_discrete(labels=0:5, "Year")ppdf("sts_common_seq.pdf", width=11, height = 7)pdev.off()```Frequency plot of top 20 sequences in STS format by race```{r,out.width="90%"}p <- ggseqfplot(mm7, proportional = F, ylabs="share", ranks=1:20, group=race)+    theme(axis.text.y = element_text(size=7))+    scale_x_discrete(labels=0:5, "Year")ppdf("sts_common_seq_race.pdf", width=11, height = 7)pdev.off()```Frequency of top 20 sequences in DSS format```{r}seqtab(seqdss(mm7), idxs = 1:20, format="STS")```Count up all the DSS format sequences```{r}n <- seqtab(seqdss(mm7), idxs = 1:300, format="STS")nrow(n)rm(n)```Frequency plot of top 20 DSS sequences```{r,out.width="90%"}p <- ggseqfplot(seqdss(mm7), proportional = F, ylabs="share", ranks=1:20)+    theme(axis.text.y = element_text(size=9.5))+    theme(axis.text.x = element_text(size=0))+    theme(legend.position = "bottom", legend.justification = c("left"))+    guides(fill=guide_legend(ncol=3))+    theme(legend.text=element_text(size=13))+    scale_x_discrete("State Transitions")ppdf("dss_common_seq.pdf", width=10, height = 7)pdev.off()```Frequency plot of top 20 DSS sequences by race```{r,out.width="90%"}p <- ggseqfplot(seqdss(mm7), proportional = F, ylabs="share", ranks=1:20, group=race)+    theme(axis.text.y = element_text(size=9.5))+    theme(strip.text.x = element_text(size = 12))+    theme(legend.position = "bottom", legend.justification = c("center"))+    guides(fill=guide_legend(ncol=7))+     theme(legend.text=element_text(size=13))+     scale_x_discrete(limits=c(1,2,3), "State Transitions")ppdf("dss_common_seq_race.pdf", width=12, height = 8)pdev.off()```Time plot by race```{r, out.width="90%"}p <- ggseqmtplot(mm7, group=race, facet_ncol=4)+    ylim(0,4)+    theme_bw()+    labs(fill="")+    theme(axis.text.x = element_text(angle = 90, vjust = 0.5, hjust=1, size=13),          strip.background = element_rect(colour="white",                                        fill="white"))+        theme(axis.text.y = element_text(size=9))+    theme(strip.text.x = element_text(size = 11))+    theme(legend.position = "bottom", legend.justification = c("center" )) +    guides(fill=guide_legend(ncol=7))+    theme(legend.text=element_text(size=13))ppdf("time_plot_race.pdf", width=16, height = 7)pdev.off()``````{r}p <- ggseqmtplot(mm7, group=race, facet_ncol=4)+    ylim(0,4)+    theme_bw()+    labs(fill="")+    theme(axis.text.x = element_text(angle = 90, vjust = 0.5, hjust=1, size=13),          strip.background = element_rect(colour="white",                                        fill="white"))+    theme(axis.text.y = element_text(size=13), axis.title=element_text(size=13))+    theme(strip.text.x = element_text(size = 15))+    guides(fill=F)+    scale_x_discrete(labels=c("None","Diabetes","Heart","Stroke","MI","Diabetes+Heart",                               "Diabetes+MI","Diabetes+Stroke","Heart+MI","Heart+Stroke",                               "MI+Stroke","Diabetes+Heart+MI","Diabetes+Heart+Stroke","Diabetes+MI+Stroke",                               "Heart+MI+Stroke","Diabetes+Heart+MI+Stroke","Death"))ppdf("time_plot_race2.pdf", width=16, height = 7)pdev.off()```-------------------------------------------------------------------------------CREATE CLUSTERS WITH DEATH AS STATE-------------------------------------------------------------------------------create substitution cost matrix with constant =1```{r}smat <- seqsubm(mm7, method="CONSTANT", cval =1) ```calculate distances using OM with indel=1 and matrix from previous step```{r}dist_mm7 <- seqdist(mm7, method = "OM", indel = 1, sm = smat)```cluster via ward```{r}w_mm7 <- hclust(as.dist(dist_mm7), method="ward.D")```goodness of fit for ward```{r}library(WeightedCluster)ward_mm7 <- as.clustrange(w_mm7, dist_mm7, ncluster=15)ward_mm7plot(ward_mm7, norm="zscore", stat=c("ASW", "HG", "PBC", "CH"))```plot state sequences with six clusters```{r, out.width="90%"}library(colorspace)library(viridis)library(ggseqplot)ggseqiplot(mm7, group=ward_mm7$clustering$cluster6, border=F, sortv="from.start")+         scale_x_discrete(labels=0:5, "Year")```plot state sequences with seven clusters```{r, out.width="90%"}ggseqiplot(mm7, group=ward_mm7$clustering$cluster7, border=F, sortv="from.start")+         scale_x_discrete(labels=0:5, "Year")```plot state sequences with eight clusters```{r, out.width="90%"}ggseqiplot(mm7, group=ward_mm7$clustering$cluster8, border=F, sortv="from.start")+         scale_x_discrete(labels=0:5, "Year")```Extract cluster solutions and add to data frame```{r}nhats_mm7$ward7 <- ward_mm7$clustering$cluster7nhats_mm7$ward8 <- ward_mm7$clustering$cluster8```Eight seems to be the best, both in terms of fit statistics and interpretability, so go with thatFix cluster variable labels and order so that it works better in plots and tables```{r}nhats_mm7 <- nhats_mm7 %>%     mutate(clus8=fct_recode(ward8,                            "Heart Disease Only"="1",                            "Diabetes Only"="2",                            "Mortality"="3",                            "No Cardiometabolic Disease"="4",                            "MI Only"="5",                            "Incident CVD with MM"="6",                            "Diabetes MM"="7",                            "Stroke Only"="8"),           clus8=fct_relevel(clus8,                             "No Cardiometabolic Disease",                             "Diabetes Only",                             "Heart Disease Only",                             "MI Only",                             "Stroke Only",                             "Incident CVD with MM",                             "Diabetes MM",                             "Mortality"))``````{r,out.width="90%"}p <- ggseqiplot(mm7, group=nhats_mm7$clus8, border=F, sortv="from.start", facet_ncol=2)+         scale_x_discrete(labels=0:5, "Year" ) +     ylab("Cumulative number of sequences")+    theme(axis.text.y = element_text(size=10),          axis.title.y = element_text(size=13),          axis.title.x = element_text(size=13))+    theme(strip.text.x = element_text(size = 13))+    theme(legend.position = "bottom", legend.justification = c("center" )) +    guides(fill=guide_legend(ncol=5))+     theme(legend.text=element_text(size=13))ppdf("cluster_solution.pdf", width=10, height = 12)pdev.off()```Plot chronograph with eight clusters```{r, out.width="90%"}p <- ggseqdplot(mm7, group=nhats_mm7$clus8, border=F, facet_ncol=2)+         scale_x_discrete(labels=0:5, "Year" ) +        theme(axis.text.y = element_text(size=10),axis.title.y = element_text(size=12))+    theme(strip.text.x = element_text(size = 13))+    theme(legend.position = "bottom", legend.justification = c("center" )) +    guides(fill=guide_legend(ncol=5))+    theme(legend.text=element_text(size=13))ppdf("chronograph_clusters.pdf", width=10, height = 12)pdev.off()    ``` time plot with 8 clusters```{r, out.width="90%"}p <- ggseqmtplot(mm7, group=nhats_mm7$clus8, facet_ncol=3)+    ylim(0,6)+    theme_bw()+    labs(fill="")+    theme(axis.text.x = element_text(angle = 90, vjust = 0.5, hjust=1, size=13),          strip.background = element_rect(colour="white",                                        fill="white"))+        theme(axis.text.y = element_text(size=12))+    theme(strip.text.x = element_text(size = 14))+    theme(legend.position = "bottom", legend.justification = c("center" )) +    guides(fill=guide_legend(ncol=9))+     theme(legend.text=element_text(size=13))ppdf("time_plot.pdf", width=14, height = 9)pdev.off()    ``````{r}p <- ggseqmtplot(mm7, group=nhats_mm7$clus8, facet_ncol=3)+    ylim(0,6)+    theme_bw()+    labs(fill="")+    theme(axis.text.x = element_text(angle = 90, vjust = 0.5, hjust=1, size=13),          strip.background = element_rect(colour="white",                                        fill="white"))+    theme(axis.text.y = element_text(size=13), axis.title=element_text(size=13))+    theme(strip.text.x = element_text(size = 14))+    guides(fill=F)+    scale_x_discrete(labels=c("None","Diabetes","Heart","Stroke","MI","Diabetes+Heart",                               "Diabetes+MI","Diabetes+Stroke","Heart+MI","Heart+Stroke",                               "MI+Stroke","Diabetes+Heart+MI","Diabetes+Heart+Stroke","Diabetes+MI+Stroke",                               "Heart+MI+Stroke","Diabetes+Heart+MI+Stroke","Death"))ppdf("time_plot2.pdf", width=14, height = 9)pdev.off()```To get the breakdown of sequences in the clusters, you have to use filter and then create new sequence objects on the subset data```{r}sub_w6 <- nhats_mm7 %>%     filter(clus8=="Incident CVD with MM")w6test <- seqdef(sub_w6,              var=2:7,              alphabet = c("none","diab","hrt","stk","mi","diab/hrt",                               "diab/mi","diab/stk","hrt/mi","hrt/stk",                               "mi/stk","diab/hrt/mi","diab/hrt/stk","diab/mi/stk",                               "hrt/mi/stk","diab/hrt/mi/stk","death"),              labels = c("None","Diabetes","Heart","Stroke","MI","Diabetes+Heart",                               "Diabetes+MI","Diabetes+Stroke","Heart+MI","Heart+Stroke",                               "MI+Stroke","Diabetes+Heart+MI","Diabetes+Heart+Stroke","Diabetes+MI+Stroke",                               "Heart+MI+Stroke","Diabetes+Heart+MI+Stroke","Death"))attributes(w6test)$cpal <- colors1``````{r}seqtab(w6test, idxs = 1:20)``````{r}seqtab(seqdss(w6test), idxs = 1:500, format="STS")```Frequency of top 20 DSS sequences in cluster #6```{r,out.width="90%"}p <- ggseqfplot(seqdss(w6test), proportional = F, ylabs="share", ranks=1:20)+    theme(axis.text.y = element_text(size=11))+    theme(axis.text.x = element_text(size=0))+    theme(legend.position = "bottom", legend.justification = c("left"))+    guides(fill=guide_legend(ncol=6))+    theme(legend.text=element_text(size=12))+    ggtitle("Incident CVD with MM")+    theme(title = element_text(size=14))ppdf("acute_cluster_common_seq.pdf", width=11, height = 7)pdev.off()```And here for cluster 7```{r}sub_w7 <- nhats_mm7 %>%     filter(clus8=="Diabetes MM")w7test <- seqdef(sub_w7,              var=2:7,              alphabet = c("none","diab","hrt","stk","mi","diab/hrt",                               "diab/mi","diab/stk","hrt/mi","hrt/stk",                               "mi/stk","diab/hrt/mi","diab/hrt/stk","diab/mi/stk",                               "hrt/mi/stk","diab/hrt/mi/stk","death"),              labels = c("None","Diabetes","Heart","Stroke","MI","Diabetes+Heart",                               "Diabetes+MI","Diabetes+Stroke","Heart+MI","Heart+Stroke",                               "MI+Stroke","Diabetes+Heart+MI","Diabetes+Heart+Stroke","Diabetes+MI+Stroke",                               "Heart+MI+Stroke","Diabetes+Heart+MI+Stroke","Death"))attributes(w7test)$cpal <- colors1``````{r}seqtab(w7test, idxs = 1:20)``````{r}seqtab(seqdss(w7test), idxs = 1:500, format="STS")```Frequency of top 20 DSS sequences in cluster 7```{r,out.width="90%"}library(ggpubr)q <- ggseqfplot(seqdss(w7test), proportional = F, ylabs="share", ranks=1:20)+    theme(axis.text.y = element_text(size=11))+     theme(axis.text.x = element_text(size=0))+    theme(legend.position = "bottom", legend.justification = c("center"))+    guides(fill=guide_legend(ncol=6))+    theme(legend.text=element_text(size=12))+    ggtitle("Diabetes MM")+    theme(title = element_text(size=14))+    theme(axis.title.y=element_blank())qpdf("diabetes_mm_cluster_common_seq.pdf", width=12, height = 7)qdev.off()r <- ggarrange(p,q, common.legend = TRUE, legend = "bottom")pdf("combined_mm_cluster_common_seq.pdf", width=11, height = 8)r +     theme(legend.position = "bottom", legend.justification = "center")dev.off()```###AND OTHER CLUSTERS SEQUENCES**DIABETES ONLY**```{r}sub_w2 <- nhats_mm7 %>%     filter(clus8=="Diabetes Only")w2test <- seqdef(sub_w2,              var=2:7,              alphabet = c("none","diab","hrt","stk","mi","diab/hrt",                               "diab/mi","diab/stk","hrt/mi","hrt/stk",                               "mi/stk","diab/hrt/mi","diab/hrt/stk","diab/mi/stk",                               "hrt/mi/stk","diab/hrt/mi/stk","death"),              labels = c("None","Diabetes","Heart","Stroke","MI","Diabetes+Heart",                               "Diabetes+MI","Diabetes+Stroke","Heart+MI","Heart+Stroke",                               "MI+Stroke","Diabetes+Heart+MI","Diabetes+Heart+Stroke","Diabetes+MI+Stroke",                               "Heart+MI+Stroke","Diabetes+Heart+MI+Stroke","Death"))attributes(w2test)$cpal <- colors1``````{r}seqtab(seqdss(w2test), idxs = 1:500, format="STS")```**HEART DISEASE ONLY**```{r}sub_w3 <- nhats_mm7 %>%     filter(clus8=="Heart Disease Only")w3test <- seqdef(sub_w3,              var=2:7,              alphabet = c("none","diab","hrt","stk","mi","diab/hrt",                               "diab/mi","diab/stk","hrt/mi","hrt/stk",                               "mi/stk","diab/hrt/mi","diab/hrt/stk","diab/mi/stk",                               "hrt/mi/stk","diab/hrt/mi/stk","death"),              labels = c("None","Diabetes","Heart","Stroke","MI","Diabetes+Heart",                               "Diabetes+MI","Diabetes+Stroke","Heart+MI","Heart+Stroke",                               "MI+Stroke","Diabetes+Heart+MI","Diabetes+Heart+Stroke","Diabetes+MI+Stroke",                               "Heart+MI+Stroke","Diabetes+Heart+MI+Stroke","Death"))attributes(w3test)$cpal <- colors1``````{r}seqtab(seqdss(w3test), idxs = 1:500, format="STS")```**MI ONLY**```{r}sub_w4 <- nhats_mm7 %>%     filter(clus8=="MI Only")w4test <- seqdef(sub_w4,              var=2:7,              alphabet = c("none","diab","hrt","stk","mi","diab/hrt",                               "diab/mi","diab/stk","hrt/mi","hrt/stk",                               "mi/stk","diab/hrt/mi","diab/hrt/stk","diab/mi/stk",                               "hrt/mi/stk","diab/hrt/mi/stk","death"),              labels = c("None","Diabetes","Heart","Stroke","MI","Diabetes+Heart",                               "Diabetes+MI","Diabetes+Stroke","Heart+MI","Heart+Stroke",                               "MI+Stroke","Diabetes+Heart+MI","Diabetes+Heart+Stroke","Diabetes+MI+Stroke",                               "Heart+MI+Stroke","Diabetes+Heart+MI+Stroke","Death"))attributes(w4test)$cpal <- colors1``````{r}seqtab(seqdss(w4test), idxs = 1:500, format="STS")```**STROKE ONLY**```{r}sub_w5 <- nhats_mm7 %>%     filter(clus8=="Stroke Only")w5test <- seqdef(sub_w5,              var=2:7,              alphabet = c("none","diab","hrt","stk","mi","diab/hrt",                               "diab/mi","diab/stk","hrt/mi","hrt/stk",                               "mi/stk","diab/hrt/mi","diab/hrt/stk","diab/mi/stk",                               "hrt/mi/stk","diab/hrt/mi/stk","death"),              labels = c("None","Diabetes","Heart","Stroke","MI","Diabetes+Heart",                               "Diabetes+MI","Diabetes+Stroke","Heart+MI","Heart+Stroke",                               "MI+Stroke","Diabetes+Heart+MI","Diabetes+Heart+Stroke","Diabetes+MI+Stroke",                               "Heart+MI+Stroke","Diabetes+Heart+MI+Stroke","Death"))attributes(w5test)$cpal <- colors1``````{r}seqtab(seqdss(w5test), idxs = 1:500, format="STS")```**MORTALITY**```{r}sub_w8 <- nhats_mm7 %>%     filter(clus8=="Mortality")w8test <- seqdef(sub_w8,              var=2:7,              alphabet = c("none","diab","hrt","stk","mi","diab/hrt",                               "diab/mi","diab/stk","hrt/mi","hrt/stk",                               "mi/stk","diab/hrt/mi","diab/hrt/stk","diab/mi/stk",                               "hrt/mi/stk","diab/hrt/mi/stk","death"),              labels = c("None","Diabetes","Heart","Stroke","MI","Diabetes+Heart",                               "Diabetes+MI","Diabetes+Stroke","Heart+MI","Heart+Stroke",                               "MI+Stroke","Diabetes+Heart+MI","Diabetes+Heart+Stroke","Diabetes+MI+Stroke",                               "Heart+MI+Stroke","Diabetes+Heart+MI+Stroke","Death"))attributes(w8test)$cpal <- colors1``````{r}seqtab(seqdss(w8test), idxs = 1:500, format="STS")```**BASELINE DIABETES**```{r}base <- nhats_mm7 %>%     filter(r6=="death")base <- seqdef(base,              var=2:7,              alphabet = c("none","diab","hrt","stk","mi","diab/hrt",                               "diab/mi","diab/stk","hrt/mi","hrt/stk",                               "mi/stk","diab/hrt/mi","diab/hrt/stk","diab/mi/stk",                               "hrt/mi/stk","diab/hrt/mi/stk","death"),              labels = c("None","Diabetes","Heart","Stroke","MI","Diabetes+Heart",                               "Diabetes+MI","Diabetes+Stroke","Heart+MI","Heart+Stroke",                               "MI+Stroke","Diabetes+Heart+MI","Diabetes+Heart+Stroke","Diabetes+MI+Stroke",                               "Heart+MI+Stroke","Diabetes+Heart+MI+Stroke","Death"))attributes(base)$cpal <- colors1``````{r}seqtab(seqdss(base), idxs = 1:500, format="STS")```
