# Supplementary material for: Longitudinal sequencing of cardiometabolic multimorbidity among older adults and association with subsequent dementia onset
Source: PLoS One. 2025 Jul 10;20(7):e0326309. doi: 10.1371/journal.pone.0326309 (PMC12244708; doi:10.1371/journal.pone.0326309)
Supplement: S1 Table — (DOCX) [file pone.0326309.s007.docx]

**Supplemental Table 1: Twenty most frequently occurring cardiometabolic disease sequences in distinctive successive state (DSS) format**

| **Sequence** | **Frequency** | **Percent** |
| --- | --- | --- |
| None | 2069 | 41.75 |
| Diabetes | 578 | 11.66 |
| None → Death | 415 | 8.37 |
| Heart | 197 | 3.97 |
| None → Heart Disease | 172 | 3.47 |
| Diabetes → Death | 133 | 2.68 |
| Mi | 120 | 2.42 |
| None → Diabetes | 117 | 2.36 |
| Heart Disease→ Death | 104 | 2.10 |
| Stroke | 104 | 2.10 |
| None → Stroke | 90 | 1.82 |
| None → MI | 79 | 1.59 |
| Diabetes → Diabetes + Heart Disease | 64 | 1.29 |
| MI → Death | 61 | 1.23 |
| Stroke → Death | 49 | 0.99 |
| Mi → Heart Disease + MI | 38 | 0.77 |
| None → Heart Disease → Death | 37 | 0.75 |
| Diabetes → Diabetes + Stroke | 34 | 0.69 |
| None → Heart Disease + MI | 28 | 0.56 |
| Heart → Diabetes + Heart Disease | 22 | 0.44 |
