# Supplementary material for: Longitudinal sequencing of cardiometabolic multimorbidity among older adults and association with subsequent dementia onset
Source: PLoS One. 2025 Jul 10;20(7):e0326309. doi: 10.1371/journal.pone.0326309 (PMC12244708; doi:10.1371/journal.pone.0326309)
Supplement: S2 Table — (DOCX) [file pone.0326309.s008.docx]

**Supplemental Table 2. Discrete time survival analysis of time to dementia onset by cardiometabolic sequence cluster**

| **Characteristic** | **HR** | **95% CI** | **p-value** |
| --- | --- | --- | --- |
| **Cluster** |  |  |  |
| *No Cardiometabolic Disease* | — | — |  |
| *Diabetes Only* | 1.25 | 1.00, 1.55 | 0.047 |
| *Heart Disease Only* | 0.91 | 0.65, 1.25 | 0.59 |
| *MI Only* | 1.41 | 0.97, 1.97 | 0.059 |
| *Stroke Only* | 1.30 | 0.85, 1.89 | 0.20 |
| *Incident CVD with MM* | 1.32 | 1.04, 1.67 | 0.022 |
| *Diabetes MM* | 1.88 | 1.44, 2.44 | <0.001 |
| **Age** | 1.11 | 1.09, 1.12 | <0.001 |
| **Race/Ethnicity** |  |  |  |
| *Non-Hispanic White* | — | — |  |
| *Non-Hispanic Black* | 1.51 | 1.25, 1.81 | <0.001 |
| *Hispanic* | 1.49 | 1.07, 2.02 | 0.014 |
| **Female** |  |  |  |
| *Male* | — | — |  |
| *Female* | 0.92 | 0.77, 1.10 | 0.36 |
| **Educational Attainment** |  |  |  |
| *High school graduate* | — | — |  |
| *Less than high school graduate* | 1.43 | 1.16, 1.76 | <0.001 |
| *Some college* | 0.95 | 0.75, 1.19 | 0.66 |
| *College graduate* | 1.01 | 0.81, 1.26 | 0.94 |
| **Income Quartile** |  |  |  |
| *Q4* | — | — |  |
| *Q3* | 1.07 | 0.84, 1.36 | 0.60 |
| *Q2* | 1.32 | 1.02, 1.71 | 0.036 |
| *Q1* | 1.71 | 1.29, 2.26 | <0.001 |
| **Married/Partnered** |  |  |  |
| *No* | — | — |  |
| *Yes* | 0.94 | 0.78, 1.14 | 0.55 |
| **Non-Cardiometabolic Conditions** | 1.07 | 0.99, 1.14 | 0.072 |
| **Entry** |  |  |  |
| *2011* | — | — |  |
| *2015* | 0.94 | 0.78, 1.14 | 0.56 |
| **Period** | 1.15 | 1.11, 1.19 | <0.001 |
| HR = Hazard Ratio, CI = Confidence Interval  MI = Myocardial infarction; CVD = Cardiovascular disease; MM = Multimorbidity | | | |
